# Active site diversification of a non‐canonical amino acid decarboxylase by merging substrate multiplexed screening with computationally guided recombination

**DOI:** 10.1002/pro.70356

**Published:** 2025-10-22

**Authors:** Allwin D. McDonald, Jonathan M. Ellis, Lydia Steger‐Wilson, Meghan E. Campbell, Andrew R. Buller

**Affiliations:** ^1^ Department of Chemistry University of Wisconsin–Madison Madison Wisconsin USA; ^2^ Department of Biochemistry University of Wisconsin–Madison Madison Wisconsin USA

**Keywords:** biocatalysis, directed evolution, fitness landscape, non‐canonical amino acid, protein engineering, pyridoxal phosphate, recombination, substrate multiplexed screening, tryptamine

## Abstract

Recombination of active site mutations is a powerful strategy to alter enzyme activity. The vastness of sequence space, however, often limits screening‐based engineering to single and double site libraries. Here, we explore focused recombination across five positions that enclose the active site of a tryptophan (Trp) decarboxylase. Our goal was to maximize the sequence diversity of enzymes that decarboxylate non‐canonical amino acids (ncAAs) with minimal screening effort. We used substrate‐multiplexed screening (SUMS) to distinguish recombinants that have impaired activity with all substrates from those that have altered specificity. In this way, we identified a larger fraction of active sequence space than could be found by single substrate screening alone. Wild‐type primer doping during library assembly enabled the enrichment of double and triple mutants while simultaneously scanning five positions. A small screening effort, <200 measurements, was sufficient to train a logistic regression model that enriched active regions of the recombination space. This iterative strategy to library design resulted in TDC variants with distinct promiscuity profiles, and one variant displayed a nearly 500‐fold increase in catalytic efficiency compared to wild‐type TDC. These results illustrate how SUMS can be combined with iterative, deep recombination to generate a panel of catalytically diverse active site architectures.

## 1 INTRODUCTION

**FIGURE 1:**
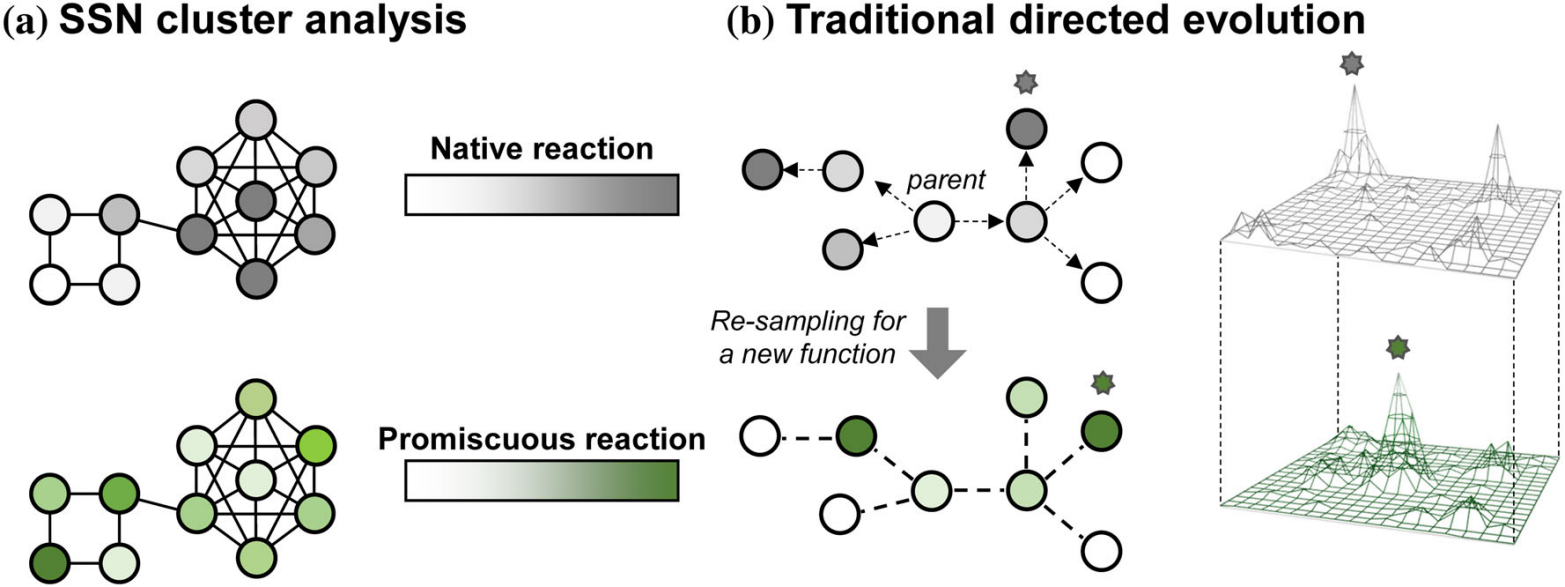
Strategies to access diverse and active enzyme variants. (a) Sequence‐similarity network (SSN) analysis identifies related sequences whose native activities vary under screening conditions (gray). This collection may be a starting point to identify variants with high activity for some new, related function of interest that was not under natural selective pressure (green) (b) Sequence space may instead be explored through directed evolution. Directed evolution to improve activity of one target reaction (gray) may serendipitously generate variants with higher activity for new reactions that were not under selective pressure (green).

Biocatalysis research often begins by screening for a desired activity among a collection of enzymes with related active sites. These collections are currently generated through two methods. One is to screen homologous natural enzymes (Figure 1a). (Baker Dockrey et al., 2018; Kelly et al., 2020; Wu et al., 2021) An alternative strategy is to tap into sequence diversity generated through directed evolution, which yields collections of sequences with properties that are distinct from naturally occurring enzymes (Almhjell et al., 2024; Chen & Arnold, 2020; Romney et al., 2019; Savile et al., 2010; Villalona et al., 2023). Such enzymes are often privileged starting points for subsequent directed evolution (Figure 1b). However, many desirable enzyme functions are relatively rare in nature and relatively few enzymes have been subject to directed evolution. It would therefore be advantageous to develop methods that directly generate diverse and functional active sites. However, mutation at multiple sites simultaneously results in a large sequence space that is mostly inactive, which has limited screening‐based recombination approaches to active site diversification (Romero & Arnold, 2009).

**FIGURE 2:**
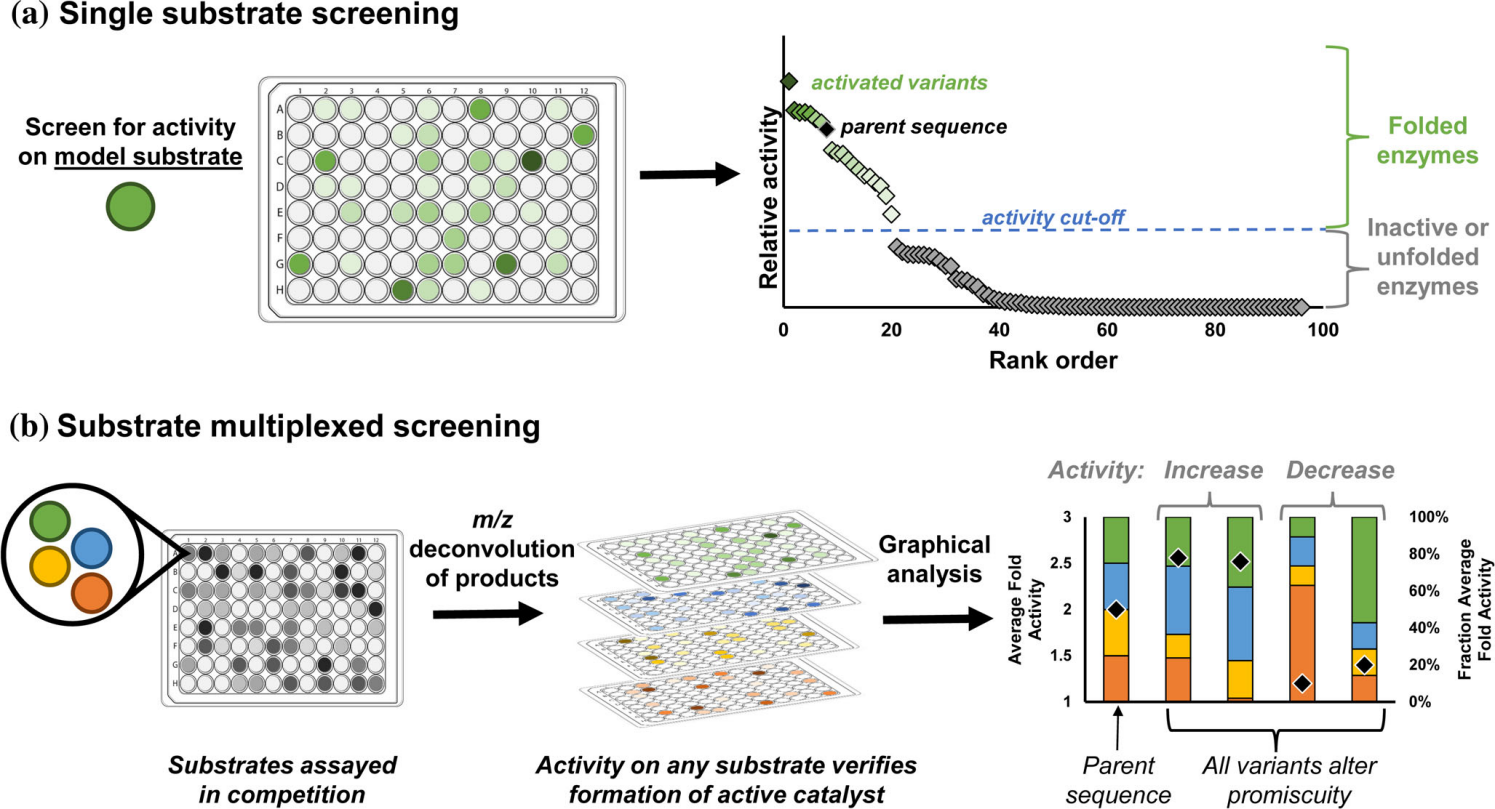
Alternate approaches for protein engineering. (a) Screening example with a single model substrate, where the goal is to identify enzyme variants with higher activity than the parent sequence. (b) Screening example in which activity on four substrates is assessed simultaneously via screening on a substrate pool. Here, activity increases or changes in promiscuity represent desirable screening outcomes.

Directed evolution is typically performed using a single model substrate (Figure 2a) (Ellis et al., 2022; Moore & Arnold, 1996; Romero & Arnold, 2009). When the biocatalysis objective is to generate a single product, this method is uniquely powerful. However, when the goal of evolution is to produce a generalist enzyme, this method can inadvertently optimize activity for the model substrate while struggling to react with substrate analogs (Almhjell et al., 2024; Amitai et al., 2007; Blikstad et al., 2014; Crawshaw et al., 2021; Liu et al., 2024; Matsumura & Ellington, 2001; Romney et al., 2019). Additionally, single substrate screening cannot distinguish mutations that simply alter soluble enzyme concentration from those that alter *k*_cat_/*K*_M_ and thereby influence catalysis itself. Parallel directed evolution to increase activity on multiple substrates is reliable, but is inefficient and seldom used (Chen et al., 2019; Reetz et al., 2005).

Previously, we demonstrated that substrate‐multiplexed screening (SUMS) can be used to engineer biocatalysts with improved activity and promiscuity (Campbell et al., 2025; McDonald, Higgins, & Buller, 2022; Villalona et al., 2023). This screening strategy places two or more substrates in direct competition, where each substrate acts as a mutual competitive inhibitor (McDonald, Higgins, & Buller, 2022). Because SUMS generates multiple, interdependent activity measurements for each variant, the corresponding structure–activity relationships are rich with biochemical information. For example, SUMS distinguishes dead enzymes from those whose activity is impaired with only a subset of substrates (Figure 2b). SUMS can also be applied to homologous enzyme panels that share a native substrate to identify natural variants with differences in promiscuous activity (Higgins et al., 2024; Zmich et al., 2023).

We hypothesized that SUMS would be well‐suited to parse distinct and functional active site architectures generated through recombination. This additional substrate activity information would increase the data density for each variant screened. We hypothesized that, with such a strategy, sparse screening data could be used to build useful computational sequence‐function models. To test this hypothesis, we considered biocatalytic decarboxylation of non‐canonical aromatic amino acids. The resulting arylethylamines represent a highly bioactive chemical space (e.g., histamine, dopamine, serotonin) (Freeman & Alder, 2002). While we and others have advanced the synthesis of aromatic ncAAs (Alfonzo et al., 2022), generation of matched decarboxylases to access their corresponding neurotransmitter analogs is a daunting protein engineering task. Long study of aromatic amino acid decarboxylases (AADCs) has shown that they have poor tolerance of substrate analogs (Runguphan et al., 2010; Torrens‐Spence et al., 2018). These results indicate that screening of broad naturally occurring homolog libraries is unlikely to yield good starting points for subsequent evolution. In 2014, Williams et al. discovered a bacterial Trp decarboxylase from *Ruminococcus gnavus* (*Rgn*TDC) that is phylogenetically distinct from the AADCs (Williams et al., 2014). We previously explored the scope of this enzyme (McDonald et al., 2019), and applied SUMS to single site libraries of *Rgn*TDC, which revealed dozens of beneficial mutations at distinct positions (McDonald, Higgins, & Buller, 2022). In particular, we observed many activated variants for 5‐substituted Trps and a few variants with improved activity for 4‐substituted Trps. We sought to further increase the activity of TDC with these challenging substrates using SUMS to expediently navigate sequence space and maintain broad promiscuity on these amino acid analogs.

## 2 RESULTS AND DISCUSSION

### 2.1 Design of substrate space

**FIGURE 3:**
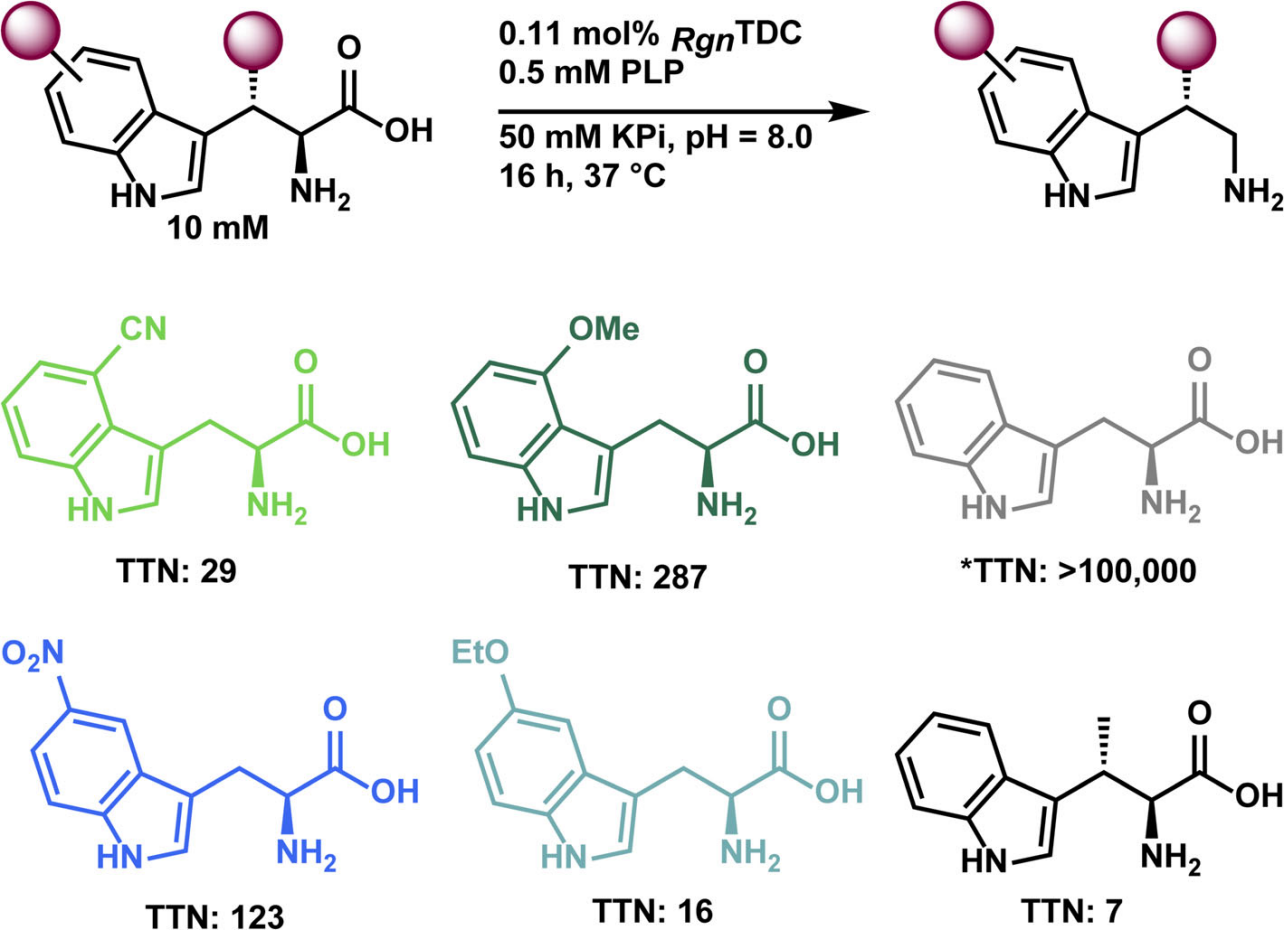
Substrates present during SUMS‐based library screening. The total turnover numbers (TTN) for wild‐type *Rgn*TDC were measured in isolation from other substrates. TTN data from Williams et al. (2014).

Substrate selection is a key initial step for the implementation of SUMS. For unimolecular transformations, substrates compete for the active site according to their independent catalytic efficiencies. When an enzyme has higher activity on some substrates in the mixture than others, low‐activity reactions can be effectively masked. Therefore, it is typically easiest to interpret the results of SUMS if the parent enzyme has a similar level of activity on each of the chosen substrates. To this end, we chose two 5‐substituted Trps that *Rgn*TDC can decarboxylate with only modest or low efficiency, 5‐NO_2_‐Trp and 5‐OEt‐Trp. It was previously observed that activity on these two substrates is not well correlated (McDonald, Higgins, & Buller, 2022), and so adding both to the substrate pool limits the possibility of missing mutations that are activating for one and not the other. The active site of RgnTDC fully encloses its substrates, and the substitution at the 4‐position is sterically demanding. We therefore also included 4‐OMe‐Trp and 4‐CN‐Trp in the mixture (Figure 3). *β*‐Me‐Trp was included to determine whether recombination could uncover gain‐of‐function mutations (<10 TON parent activity). However, throughout all screened libraries, no variants displayed observable activity with *β*‐Me‐Trp under competition, and so this product is not discussed in subsequent screening analysis.

### 2.2 Unbiased recombination of activating *Rgn*TDC mutations

**FIGURE 4:**
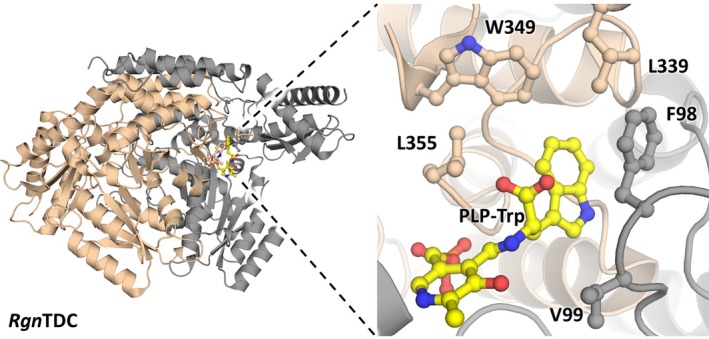
Active site of *Rgn*TDC with targeted residues for engineering. The individual monomers of the dimer (PDB: 4OBV) are shown in gray and cream, and a modeled Trp external aldimine is shown in yellow (modeled). Mutated residues are highlighted.

**FIGURE 5:**
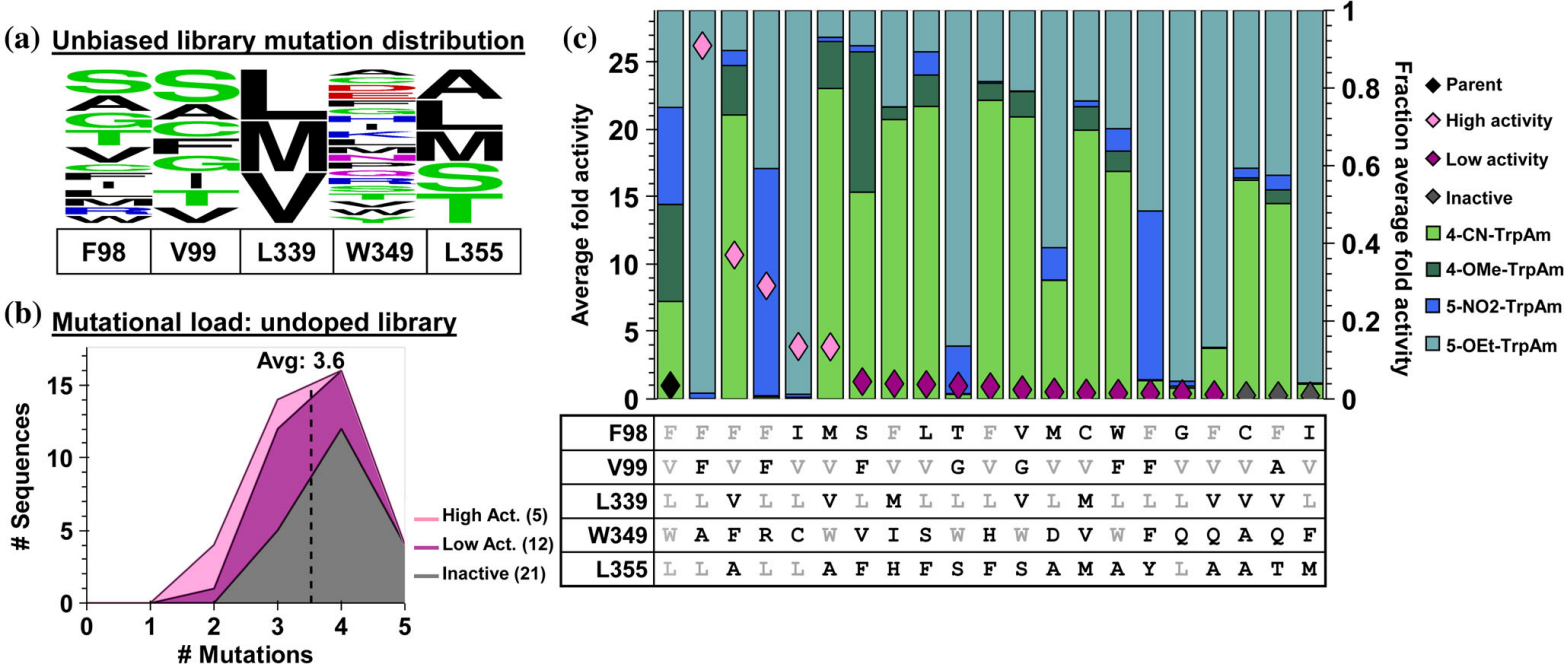
Sequence‐activity data of a naïve recombination library. (a) WebLogo depicting expected mutation distribution. (b) Mutational load distribution of unique sequences. (c) SUMS specificity profiles and mutational identity for the top 20 variants.

We targeted five active‐site residues for recombination: F98, V99, L339, W349, and L355 (Figure 4). A complete recombination space would comprise a daunting 3.2 million members, precluding comprehensive sampling through plate‐based screening. We considered a more limited space comprising mutations at each position that were previously found to be neutral or activating for one or more substituted Trps (Figure 5a). (McDonald, Higgins, & Buller, 2022) This “focused library” consisted of 28,800 possible sequences and a relatively high expected mutational rate of 4.3 mutations per clone. We used LC–MS to assay members of this library against an equimolar mixture of the selected 4‐ and 5‐substituted Trps.

We performed a small sampling of this space and acquired SUMS sequence‐function relationships for 37 unique variants (Supplementary Figure 3). We measured each substituted tryptamine product formed and calculated their ratios relative to parent, which normalizes differences in ionization efficiency and reduces plate‐to‐plate variation over the course of the study. Changes in the promiscuity of the variants can then be readily visualized by a shift in the size of the bars shown in Figure 5c. Measurement of variant “activity,” however, is more nuanced. The simplest metric, the change in total product, is easy to understand but is of limited use for the study here. For example, *small* decreases in activity with a more reactive substrate (e.g., 4‐OMe‐Trp) may obscure *large* gains in activity with a less reactive substrate (e.g., 5‐OEt‐Trp). Such a variant would represent an innovative region of sequence space that would be overlooked with analysis based on solely total product. We, therefore, averaged the fold‐activity change for the individual products, which compensates for differences in parent activity and better captures desirable changes in aggregate activity.

We divided the variants into three categories based on their activity. Variants with greater than 1.5‐average fold activity were labeled as “high activity” variants, while those with less than 0.3‐average fold activity were labeled as “inactive.” The intermediate variants and wild‐type enzyme were designated as “low activity” (Figure 5b). Only 5 variants (~14%) displayed high activity, and 12 variants (~32%) had low activity, with the remaining 21 variants (~57%) inactive (Figure 5c). Naming of these variants by their multiple mutations can be cumbersome, so abbreviations were used according to the amino acid identities at each targeted site. Hence, the most active variant, TDC F98F, V99F, L339L, W349F, and L355L has two mutations and is abbreviated TDC‐FFLAL. This variant had a large increase in activity on 5‐OEt‐Trp. In contrast to the high specificity of TDC‐FFLAL, a single mutational step to TDC‐FFLRL results in a variant with boosts in activity for *both* 5‐substituted Trps (Figure 5c). We also identified a variant with high activity on 4‐substituted Trps, TDC‐FVVFA. Notably, a handful of low activity variants exhibited dual activity with both a 4‐substituted and 5‐substituted Trp, many of which were triple and quadruple mutants. These results indicate that this mutational space represents a promising region for discovering distinct new enzymes. However, most of the sequences had low overall activity.

Analysis of the library sequencing data showed an average mutational load of 3.6 mutations per clone (Figure 5b). Notably, most sequences with four mutations and all quintuple mutants were inactive. Since innovative areas of sequence space are restricted to functional enzymes, we considered a strategy to bias the library space away from these inactive variants.

### 2.3 Primer doping to reduce mutational burden

**FIGURE 6:**
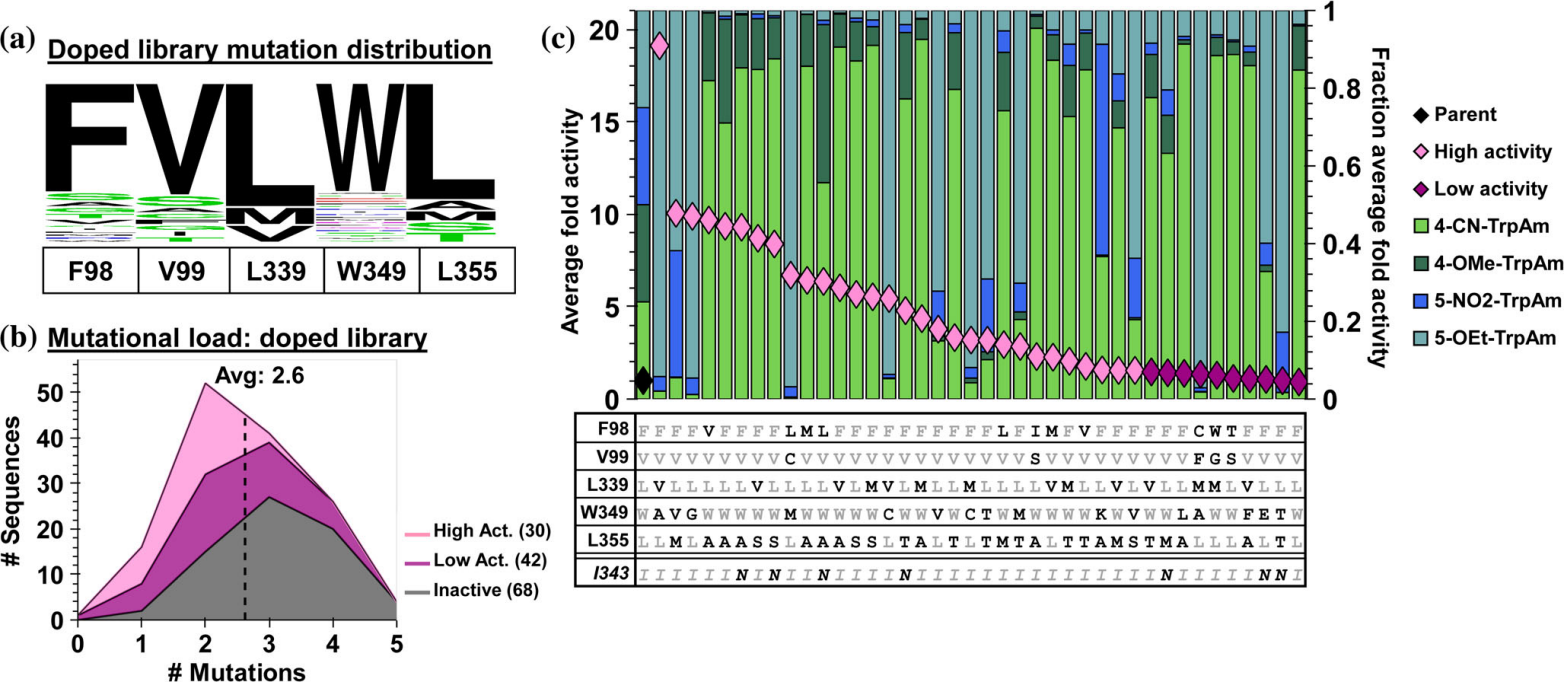
Sequence‐activity data of wild‐type primer‐doped recombination library. (a) WebLogo depicting expected mutation distribution from wild‐type primer doping. (b) Mutational load distribution of unique sequences. Note, we do not include I343N in the average, so that the active site diversity is directly comparable to previous libraries. (c) SUMS specificity profiles and mutational identity for the top 40 variants.

We re‐tuned the mutational rate by mixing primers containing the wild‐type sequence along with the mutagenic primers during PCR. This “wild‐type doping” strategy maintains the identity of the theoretical sequence space but lowers the mutational load, which we hypothesized would increase the proportion of active sequences. The primers were added in a 3:2 ratio of mutagenic to wild‐type primers, giving an expected mutational rate of 2.6 mutations per clone (Figure 6a).

Preliminary screening revealed that approximately half of the sequences in this new library were designated as active, and we therefore screened it to greater depth, collecting 140 unique variants. Histogram analysis revealed the average active site mutational rate was indeed reduced to 2.6 mutations per variant (Figure 6b). Sequencing revealed that an unintended distal mutation, I343N, was common in the library. We compared variants that differed only by the presence of I343N and observed this unintended mutation caused no consistent change in promiscuity but, serendipitously, increases overall activity (Supplementary Figure 1).

A small number of variants displayed distinct shifts in promiscuity. As before, we identified variants that were activated with just 5‐OEt‐Trp, or with both 5‐substituted Trps, but the mutations that confer these effects are wholly distinct. Across this sequence space, all variants that were activated for 5‐substituted substrates had a mutation at W349. These data also revealed a double mutant, TDC‐FVLKA, that was activating for both 4‐CN and a 5‐NO_2_ substrates. These results highlight that wild‐type primer doping can bias sequence space toward functional, generalist variants. While this library was enriched in active sequences, we observed a higher degree of homogeneity in the product distributions for this library compared to the undoped library (Figure 6c, Supplementary Figure 4). We hypothesized that the decrease in functional diversity is a direct consequence of the decreased sequence diversity. We therefore considered whether the collected sequence‐function information could be used for computational modeling to increase mutational load while maintaining enzyme function.

### 2.4 Logistic regression modeling of TDC variants

We used modeling to identify mutations that exhibited a high propensity for negative cooperativity. Our hypothesis was that the undoped library, with its high quantity of triple and quadruple mutants, would provide rich information on cooperative effects. Complementary to this data, the primer‐doped library is enriched in single and double mutants. Detailed quantification of the rich cooperativity in this sequence space would require many more data points. Instead, we pursued a classification scheme via logistic regression to identify strongly deleterious mutations.

We used one‐hot encoding due to its simplicity and prior success in other modeling endeavors (Ellis et al., 2022). We considered that there also may be general epistatic effects related to decreased thermal stability of proteins bearing many mutations that are independent of mutation identity. We therefore appended an additional binary vector of size 5 to separate effects due to the number of mutations, agnostic of mutation identity.

**FIGURE 7:**
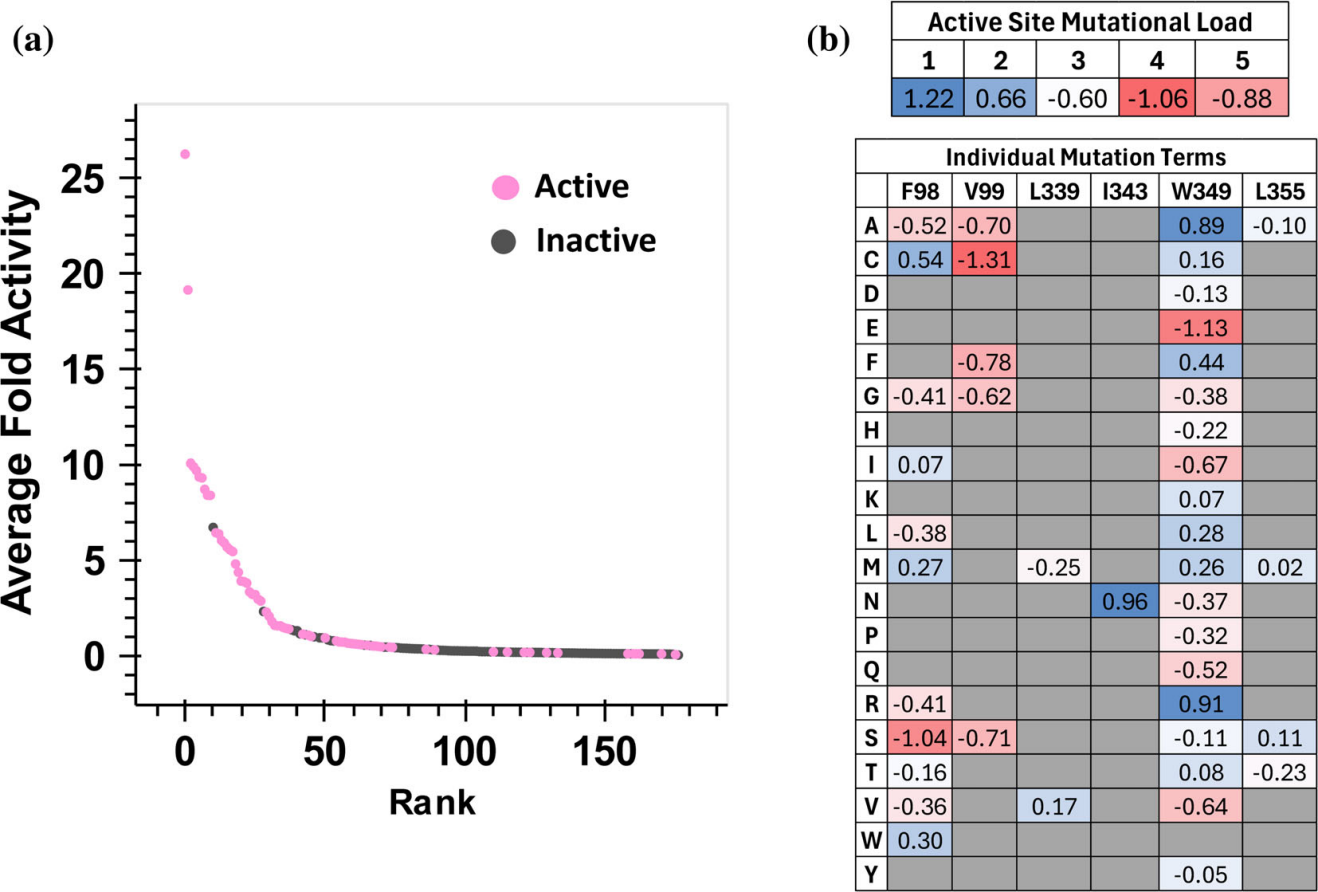
Logistic regression analysis of *Rgn*TDC active site recombination sequence space. (a) Labeling of training data by trained model. (b) Weight matrix values of logistic regression model. The upper table shows the influence of active site mutational load, separate from mutation identity. The lower table shows the influence of individual mutations on the probability of a given variant being labeled active. The model intercept optimized to a value of 0.20, corresponding to a probability value of 0.45 and a label of “active” for the unmutated parent sequence.

Area under curve (AUC) analysis with leave‐one‐out cross‐validation was used to assess model fidelity as a function of labeling threshold (Supplementary Figure 2A). A labeling threshold of 0.45 average fold activity was chosen, which includes lower activity variants as “active,” as these variants may represent innovative areas of sequence space. Application of this scheme designates 72 of the 177 unique sampled sequences as active. Receiver operating characteristic analysis was then conducted with the labeled dataset, resulting in a probability threshold of 0.48 (Supplementary Figure 2B). The resulting model had an overall accuracy of 82% (Figure 7a, Supplementary Table 1A). We considered whether model accuracy might depend on mutational load but found that the model had good predictive power across the sequence space (Supplementary Table 1B). These metrics indicate that the model has captured an accurate representation of the training data.

To determine which mutations were predicted to be strongly destabilizing, we visualized the underlying weight values of the computational model (Figure 7b). As expected, a clear negative correlation was observed with increasing mutational load. This analysis revealed many mutations that were deleterious across this recombination space. Even though mutation at V99 is beneficial in some double mutants, the presence of mutations here is generally associated with less fit variants. Additionally, F98S and W349E were predicted to be highly deleterious. We hypothesized that the removal of these mutations would reduce negative cooperative effects and increase the number of functional sequences at higher mutational loads. We note the I343N mutation is the most beneficial mutation in the weight matrix and was fixed during subsequent recombination.

### 2.5 Sampling of a computationally‐refined sequence space

**FIGURE 8:**
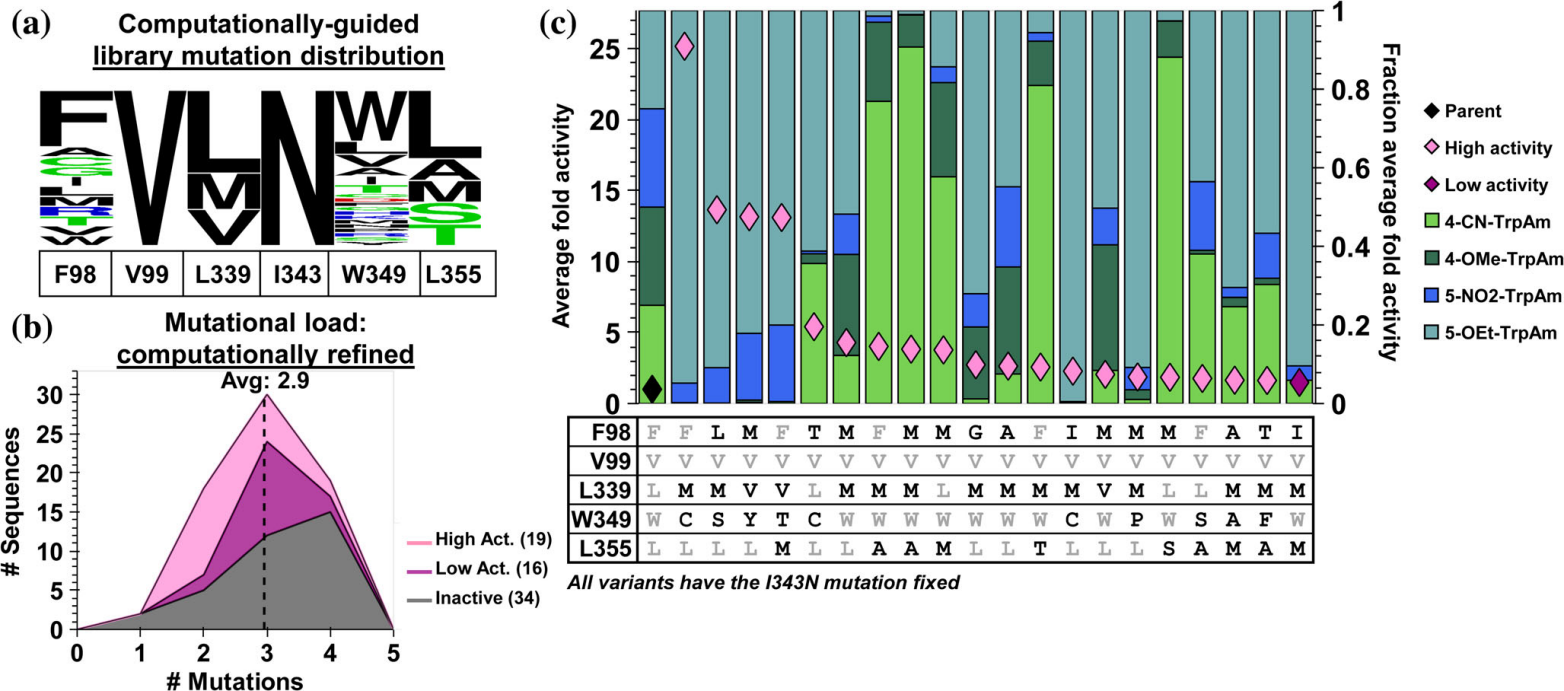
Sequence‐activity data of post‐logistic regression optimized recombination library. (a) WebLogo depicting expected mutation distribution following linear regression and wild‐type primer doping. (b) Mutational load distribution of unique sequences. Note, we do not include I343N in this calculation so that the active site diversity is directly comparable to previous libraries. (c) SUMS promiscuity profiles and mutational identity for the top 20 variants.

We implemented the mutational changes described above, resulting in a focused recombination space encompassing 2970 sequences. A library representing this space was constructed using a 7:3 ratio of mutational to wild‐type primers (Figure 8a). We acquired sequence and function data for 69 variants from this new library, which had a small increase in the average active site mutational load from 2.6 to 2.9 mutations (Figure 6b). Gratifyingly, this increase in mutational load did not compromise the fraction of the library that had high activity.

We observed significant diversity in the promiscuity profile of these variants and some trends emerged (Figure 8c). As with previous libraries, mutation at W349 is associated with high activity on 5‐substituted substrates. The largest increases in 4‐substituted Trp activity (TDC‐FVMWA, TDC‐MVMWA, TDC‐MVLWM, and TDC‐MVLWS) often had mutations at L355 while maintaining W349. Notably, more variants demonstrated generalist activity in this library. Sequence analysis of variants that are activating for both 4‐ and 5‐substituted substrates (TDC‐MVMWL, TDC‐GVMWL, TDC‐AVMWL, and TDC‐MVVWL) shows that there are no mutations at either W349 or L355, highlighting how different position around the active site can influence reactivity.

### 2.6 Assembly and single‐substrate assessment of a collection of diverse and activated variants

**FIGURE 9:**
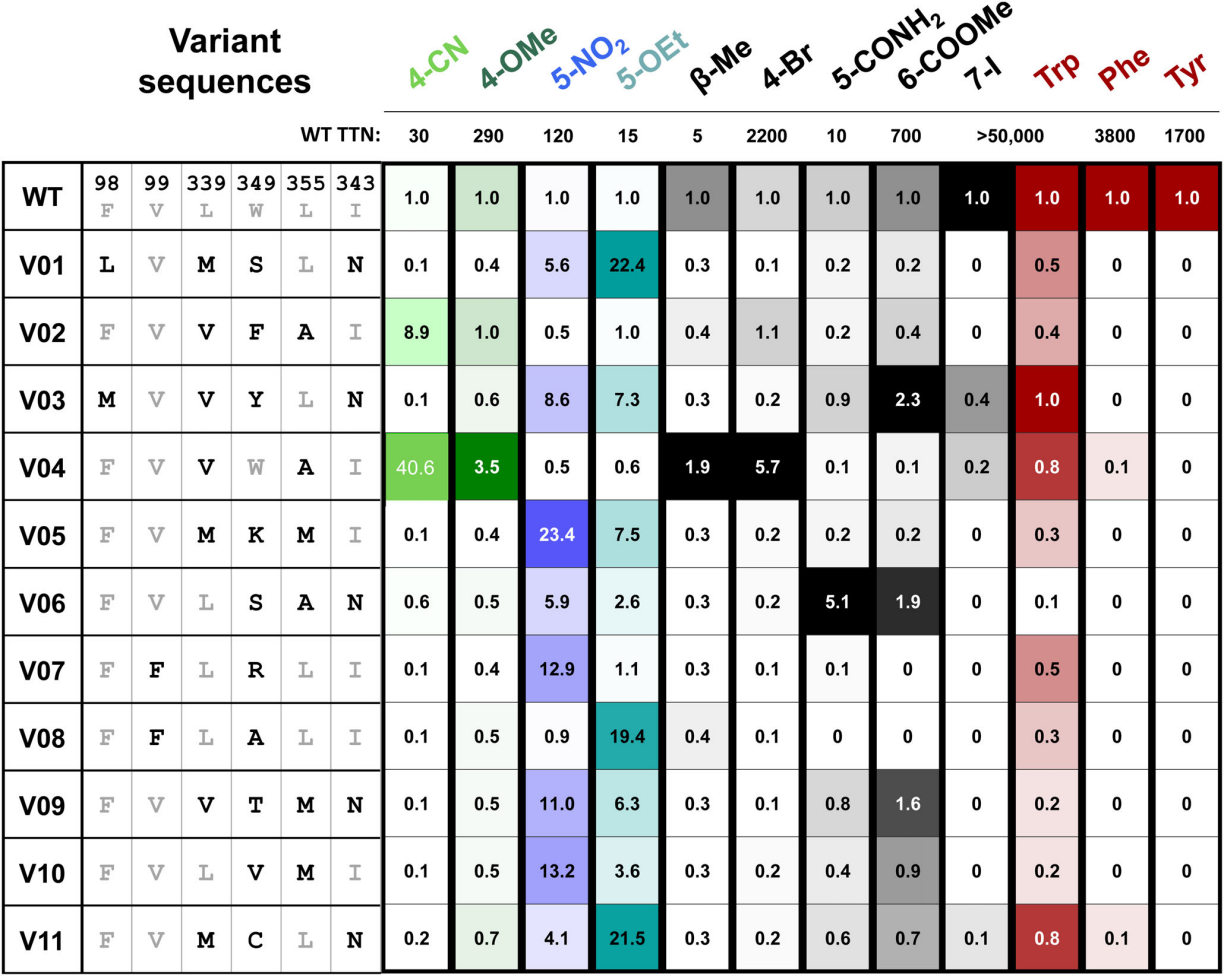
Selected variants and their fold‐activity changes with various substrates. Assays conducted with enzyme lysates and 2 mM Trp analog. See supplementary information for detailed experimental information.

We next tested the ability of the SUMS‐based recombination to identify variants with higher activity on single substrates, that is, no longer in competition. For this analysis, we curated a set of 27 diverse, activated variants. For the sake of brevity, we refer to these select TDC variants as V01–V27 and their mutations are given in Figure 9. These enzymes have an average of 2.7 mutations and were chosen based on their large boosts in activity with one or more substrates, as well as their sequence diversity (Supplementary Table 2). This set comprised 5 variants from the undoped library, 11 variants from the wild‐type primer doped library, and 11 variants from the logistic regression optimized library.

Assaying activity on single substrates may yield different results from SUMS, as there is no influence of substrate competition on apparent changes in activity. Gratifyingly, single‐substrate screening revealed many highly activated variants (Figure 9). Two variants, V02 and V04, displayed increases in activity with 4‐substituted Trps. However, V04 had the highest increases in activity with each of the 4‐substituted Trp, displaying 41‐fold and 3.5‐fold improvements in 4‐CN‐ and 4‐OMe‐tryptamine production, respectively (Figure 9, Supplementary Table 3). These increases are notable as 4‐substituted Trps are the most challenging class of substrate for the wild‐type enzyme.

As anticipated from the SUMS screening data, many variants had improved activity with 5‐substituted Trps (Figure 9). These variants all contain a mutation at W349. Many of the variants increased activity asymmetrically with the different 5‐substituted substrates. The best variant for 5‐NO_2_‐Trp is V05 (23‐fold), while the best 5‐OEt‐Trp is V01 (22‐fold). Both variants contain 3 active site mutations, with only the L339M mutation in common.

**FIGURE 10:**
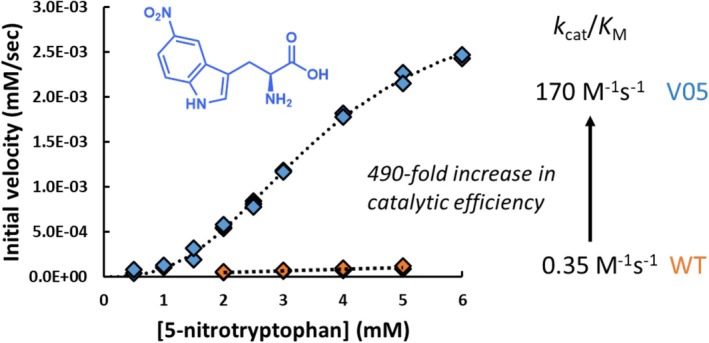
Steady‐state kinetic analysis of 5‐nitrotryptophan decarboxylation by WT and engineered TDC. Product formation with WT was first‐order in substrate across the concentration range tested. Quantitation of V05 activity indicated cooperativity between and data fit with a *k*_cat_ = 0.63 s^−1^, *K*_1/2_ = 3.6 mM, and a Hill coefficient of 2.7. Further experimental details are provided in the Supplementary Information.

We further validated the engineering results through classical Michaelis–Menten kinetic analysis with decarboxylation of 5‐NO_2_‐Trp. Kinetic analysis revealed wild‐type was indeed a feeble catalyst, with a catalytic efficiency of just 0.35 M^−1^ s^−1^ (Figure S5). In contrast, V05 had appreciable 5‐NO_2_‐Trp decarboxylation activity (Figure S6) with a catalytic efficiency of 170 M^−1^ s^−1^. While still slow compared to the rate of WT catalyzing its native reaction (6900 M^−1^ s^−1^), the three mutations in V05 confer a 490‐fold increase in catalytic efficiency (Figure 10).

### 2.7 Extrapolation of substrate space to decarboxylation of new amino acids

**FIGURE 11:**
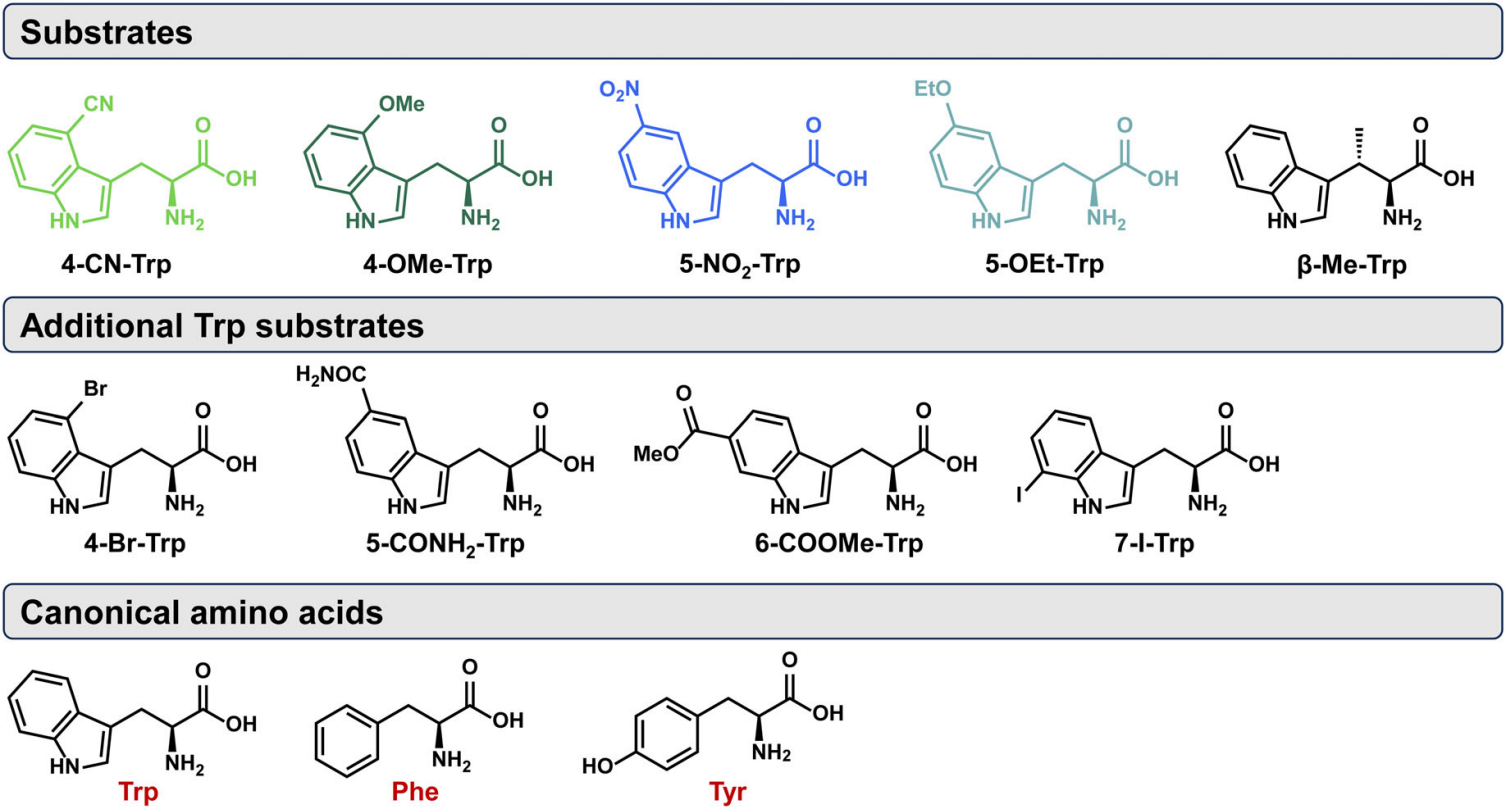
Subset of new substrates tested against consolidated *Rgn*TDC library.

The central hypothesis of this research was that the generation of diverse active sites using SUMS would serendipitously increase activity with Trp analogs, even those not included in the original assays. To screen the extrapolation potential of this library, a variety of additional substituted Trps were synthesized using previously engineered TrpB catalysts and screened (Figure 11) (Boville et al., 2018; Buller et al., 2015; Herger et al., 2016). V04, the most active variant for 4‐substituted Trps, also showed an increase in activity against 4‐Br‐Trp (5.7‐fold) and, surprisingly, *β‐*Me‐Trp (1.9‐fold, Figure 9). V06, a variant with only modest activity increases on the screened 5‐substituted Trps, was the only variant tested to show improved activity on 5‐CONH2‐Trp (5.1‐fold). This variant also demonstrated improved activity on 6‐COOMe‐Trp (1.9‐fold) alongside V03 (2.3‐fold) and V09 (1.6‐fold).

Finally, we assessed the activities of the curated library against canonical aromatic amino acids. We hypothesized that activity against endogenous amino acids would lead to increased cellular stress and reduced cell density, thus reducing enzyme titer and affording an implicit selective pressure during heterologous expression. Additionally, activity on endogenous amino acids could represent a significant source of contamination if TDC were to be deployed in a whole cell or cell lysate biocatalytic context. While the parent enzyme displayed some activity with each aromatic amino acid, all variants in this curated library displayed negligible activity with both l‐phenylalanine and l‐tyrosine, and most variants showed reduced activity against l‐Trp. These results are consistent with the omission of l‐phenylalanine and l‐tyrosine analogues during SUMS screening, permitting loss of function in this region of chemical space.

## 3 DISCUSSION

The initial motivation for this research was to identify highly active TDC variants for the generation of bioactive 4‐ and 5‐substituted tryptamines. Engineering to increase activity with any one of these substrates would itself be a straightforward, if not trivial, task. SUMS‐based recombination enabled evolution to increase activity with multiple distinct substrates *simultaneously*. This result is notable because activity between some substrates, such as the 5‐substituted Trps, is poorly cross‐correlated and no single iterative site saturation mutagenesis (ISM) effort could have resulted in the highly proficient catalysts discovered here. Indeed, we observed similar trends from our results here, with many variants only showing increased activity with one tested 4‐ or 5‐substituted Trp. Hence, SUMS‐based recombination enables evolution for multiple specialist catalysts while avoiding duplicate efforts. Happily, many variants that displayed high activity on 4‐ and 5‐substituted Trps during screening also displayed high activity with new of 4‐ and 5‐substituted Trps that were not under selection. Activity of V03 and V06 even increased activity with 6‐COOMe‐tryptophan, even though no 6‐substituted Trps were included during screening. These sequences represent new starting points for future engineering efforts or for exploration of utility in synthetic or biological contexts.

A notable feature of these variants is that many have improved properties for selective in vivo ncAA decarboxylation. That is, most variants do not react with Tyr or Phe, and even activity on the native Trp is dramatically reduced. Previous TDC engineering for activity on *β*‐hydroxy amino acids serendipitously increased activity with free Leu and Met (Mcdonald, Bruffy, et al., 2022) and Trp remained a privileged substrate. For many *Rgn*TDC variants in our present study, we observed a total loss of Trp activity while gaining activity on larger Trp analogs. This specificity shift has clear implications for in vivo TDC applications where decarboxylation of native metabolites is not desirable, such as heterologous expression in yeast (Holtz et al., 2024). Additionally, application of an orthogonal TDC that is selective for a Trp derivative may prove useful for synthetic biology.

Active site mutagenesis is a robust strategy to efficiently engineer enzyme activity (Reetz et al., 2005; Reetz & Carballeira, 2007). However, there is a tension between the size of a recombination space, which might contain highly activated sequences, and minimization of screening time. Techniques such as combinatorial active site saturation test (CASTing) (Reetz et al., 2005), ISM (Reetz & Carballeira, 2007) and focused rational iterative site‐specific mutagenesis (Li et al., 2020) have sought to collapse the large combinatorial space by iteratively fixing mutations. Here, we offer a distinct approach to navigating such sequence spaces. Rather than fixing mutations, which limits the ability to capture cooperative effects (Johnston et al., 2024), we scan over a larger combinatorial space and use a combination of experimental and computational approaches to iteratively bias the library space toward more active regions. The approach of supplementing mutational primers with those containing the wild‐type sequence is conceptually simple, but rarely reported (Goldberg et al., 2003), and allows for fine‐tuning of mutational load to mitigate sampling of defunct enzymes. The resulting sparse sequence‐function data were sufficient to train a simple logistic regression model that identifies each individual mutation's average effect on activity when in combination with other mutations. Future efforts to empower the modeling component of this research might incorporate computational predictions of enzyme stability in conjunction with more comprehensive encoding schemes such as those that describe physiochemical properties of the substrates. SUMS data also provide rich information for training more quantitative modeling strategies, such as linear or Gaussian kernel regression (Romero et al., 2013).

## 4 CONCLUSIONS

Application of a SUMS‐guided deep recombination of the *Rgn*TDC active site resulted in a diverse set of sequences with distinct activity profiles for tryptophan analogs. We used prior information from five site‐saturation mutagenesis libraries of *Rgn*TDC (96 unique sequences) to limit the theoretical recombination space to 28,800 possible variants. By analyzing screens with a single‐quad LC–MS instrument, products with distinct *m*/*z* values were quantitated in parallel with no more effort required than single‐substrate assays. Information from <200 variants was sufficient to build a logistic regression model, which further enriched the library in active sequences by pruning universally deleterious mutations (Herger et al., 2016). Additionally, we serendipitously identified an activating mutation, I343N, that is on an unresolved loop in the previous crystal structures (Williams et al., 2014). These results represent the first steps in rapid sequence space exploration using computational modeling based on SUMS data. Screening of 70 additional variants from a computationally guided library resulted in the identification of TDC variants with complementary activity profiles. In all, sequence‐function relationships for just ~250 variants, representing <1% of the total sequence space were sampled. These results underscore the utility of SUMS‐guided deep recombination as a protein engineering approach.

## 5 MATERIALS AND METHODS

See supporting information for detailed experimental materials and methods.

## AUTHOR CONTRIBUTIONS

**Allwin D. McDonald:** Conceptualization; methodology; visualization; writing – original draft; writing – review and editing; investigation; validation. **Jonathan M. Ellis:** Conceptualization; methodology; validation; software; data curation; writing – review and editing; writing – original draft; visualization; investigation. **Lydia Steger‐Wilson:** Investigation; validation; methodology. **Meghan E. Campbell:** Data curation; supervision; visualization; validation; writing – original draft; writing – review and editing. **Andrew R. Buller:** Conceptualization; investigation; funding acquisition; writing – original draft; writing – review and editing; supervision; project administration.

## CONFLICT OF INTEREST STATEMENT

A.D.M. and A.R.B. are inventors on a patent related to the synthetic application of engineered TDCs.

## Supporting information


**Supplementary Figure 1.** Ratio of fold‐activity changes for sampled variants with and without I343N. A ratio above 1 indicates that I343N confers improved activity, while a ratio below 1 indicates reduced activity. The sequence position “X” represents the 343 position.
**Supplementary Figure 2**. (A) Area under curve (AUC) analysis of various average fold‐activity labeling thresholds and corresponding percent of unique sequences labeled as active. The black line indicates the chosen labeling threshold of 0.45. (B) Receiver operating characteristic (ROC) analysis of the labeled dataset. The orange point indicates the best model prediction labeling threshold of 0.48.
**Supplementary Figure 3**. Retention of function curves of all sequenced variants for (A) naïve (undoped), (B) wild‐type primer‐doped, and (C) post‐logistic regression optimized recombination libraries.
**Supplementary Figure 4**. Mutation load influence on cumulative activity changes. (left) Observed fraction of sequenced wells with a given number of mutations in undoped (*n* = 33 wells) and doped (*n* = 226 wells) plates, including parent control wells. (Right) Kernel density estimate of the probability of a variant having a given fold change in total activity. These results show that primer doping reduces mutational load and samples a more active sequence space.
**Supplementary Figure 5**. Initial velocity measurements of wild‐type RgnTDC with 5‐NO2‐Trp. The linear relationship indicates the KM >5 mM and the slope therefore corresponds to kcat/KM.
**Supplementary Figure 6**. Example of a UPLC‐MS trace for the conversion of 5‐nitrotryptophan to 5‐nitrotryptamine.
**Supplementary Table 1**. (A) Confusion matrix and accuracy of trained logistic regression model. (B) Confusion matrices and accuracies of trained logistic regression model on various active site mutational load data subsets.
**Supplementary Table 2**. Curated library used for variant validation. Within each plate section, 2 sterile control wells (D03, F03) and 3 parent control wells (C03, E03, G03) were included. For rescreening, this layout (A01–H04) was copied two additional times (A05‐H08, A09‐H12) to fill a 96‐well plate.
**Supplementary Table 3**. Fold activities of tryptamine formation for all curated library variants (V01–V27) compared to parent when assayed against the indicated amino acid.

## Data Availability

The data that support the findings of this study are available from the corresponding author upon reasonable request.
