# Supplementary material for: Active site diversification of a non‐canonical amino acid decarboxylase by merging substrate multiplexed screening with computationally guided recombination
Source: Protein Sci. 2025 Oct 22;34(11):e70356. doi: 10.1002/pro.70356 (PMC12541894; doi:10.1002/pro.70356)
Supplement: Supplementary file 1 — Supplementary Figure 1. Ratio of fold‐activity changes for sampled variants with and without I343N. A ratio above 1 indicates that I343N confers improved activity, while a ratio below 1 indicates reduced activity. The sequence position “X” represents the 343 position. Supplementary Figure 2. (A) Area under curve (AUC) analysis of various average fold‐activity labeling thresholds and corresponding percent of unique sequences labeled as active. The black line indicates the chosen labeling threshold of 0.45. (B) Receiver operating characteristic (ROC) analysis of the labeled dataset. The orange point indicates the best model prediction labeling threshold of 0.48. Supplementary Figure 3. Retention of function curves of all sequenced variants for (A) naïve (undoped), (B) wild‐type primer‐doped, and (C) post‐logistic regression optimized recombination libraries. Supplementary Figure 4. Mutation load influence on cumulative activity changes. (left) Observed fraction of sequenced wells with a given number of mutations in undoped (n = 33 wells) and doped (n = 226 wells) plates, including parent control wells. (Right) Kernel density estimate of the probability of a variant having a given fold change in total activity. These results show that primer doping reduces mutational load and samples a more active sequence space. Supplementary Figure 5. Initial velocity measurements of wild‐type RgnTDC with 5‐NO2‐Trp. The linear relationship indicates the KM >5 mM and the slope therefore corresponds to kcat/KM. Supplementary Figure 6. Example of a UPLC‐MS trace for the conversion of 5‐nitrotryptophan to 5‐nitrotryptamine. Supplementary Table 1. (A) Confusion matrix and accuracy of trained logistic regression model. (B) Confusion matrices and accuracies of trained logistic regression model on various active site mutational load data subsets. Supplementary Table 2. Curated library used for variant validation. Within each plate section, 2 sterile control wells (D03, F03) and 3 parent cont [file PRO-34-e70356-s001.pdf]

Supplementary Information

Active site diversification of a non-canonical amino acid decarboxylase by merging substrate multiplexed screening with computationally guided recombination

Allwin D. McDonald*, Jonathan M. Ellis*, Lydia Steger-Wilson, Meghan E. Campbell, Andrew R. Buller

Correspondence to: arbuller@wisc.edu

<u>Contents:</u>

Supplementary Figures

Supplementary Tables

Supplementary Materials and Methods

- **Protein and DNA Sequences**
- **Cloning, Expression, Purification, and Storage of RgnTDC**
- **Library Construction**
- **Screening of RgnTDC libraries**
- **Logistic Regression Modelling**
- **Validation of Curated Variants**

NMR

Supplementary References

Supplementary Figures

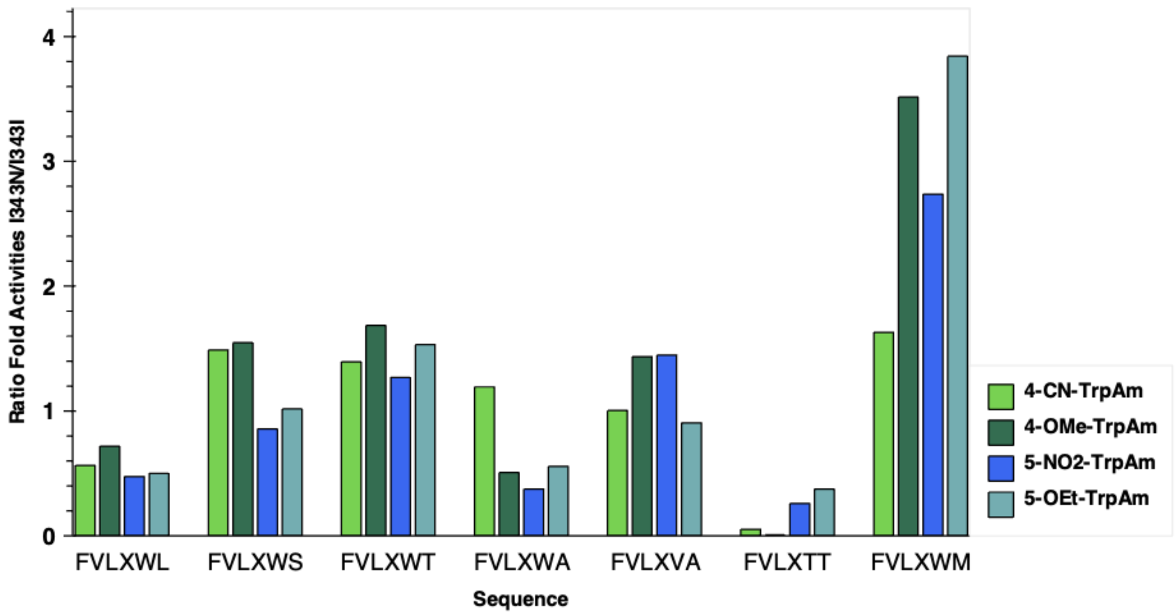

Supplementary Figure 1. Ratio of fold-activity changes for sampled variants with and without I343N. A ratio above 1 indicates that I343N confers improved activity, while a ratio below 1 indicates reduced activity. The sequence position 'X' represents the 343 position.

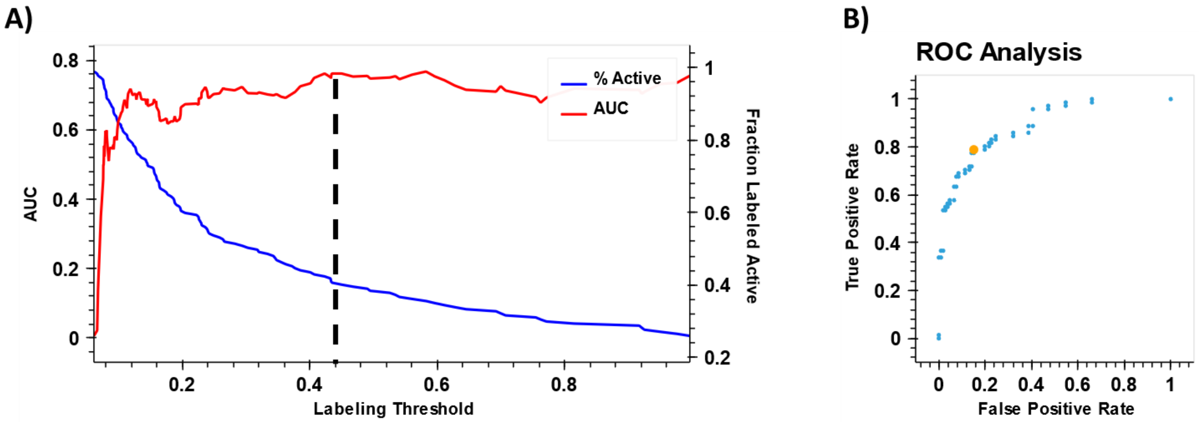

Supplementary Figure 2. A) Area under curve (AUC) analysis of various average fold-activity labeling thresholds and corresponding percent of unique sequences labeled as active. The black line indicates the chosen labeling threshold of 0.45. **B)** Receiver Operating Characteristic (ROC) analysis of the labeled dataset. The orange point indicates the best model prediction labeling threshold of 0.48.

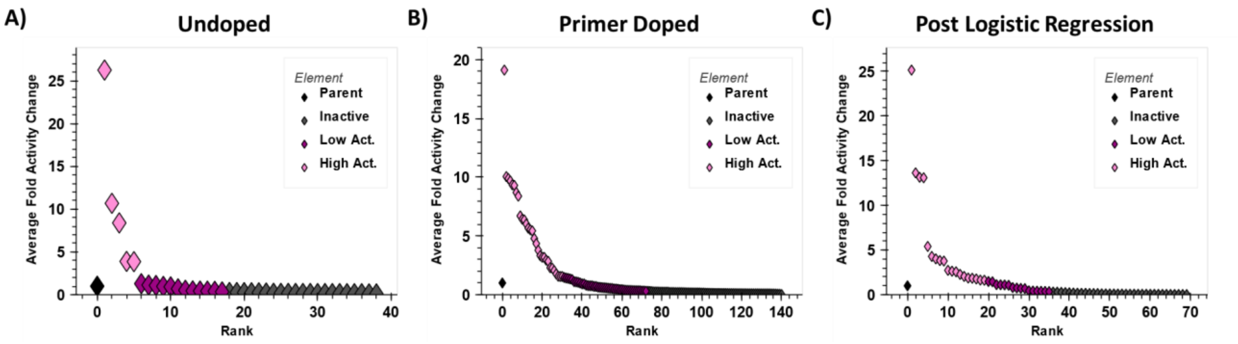

Supplementary Figure 3. Retention of function curves of all sequenced variants for **A)** naïve (undoped), **B)** wild-type primer-doped, and **C)** post-logistic regression optimized recombination libraries.

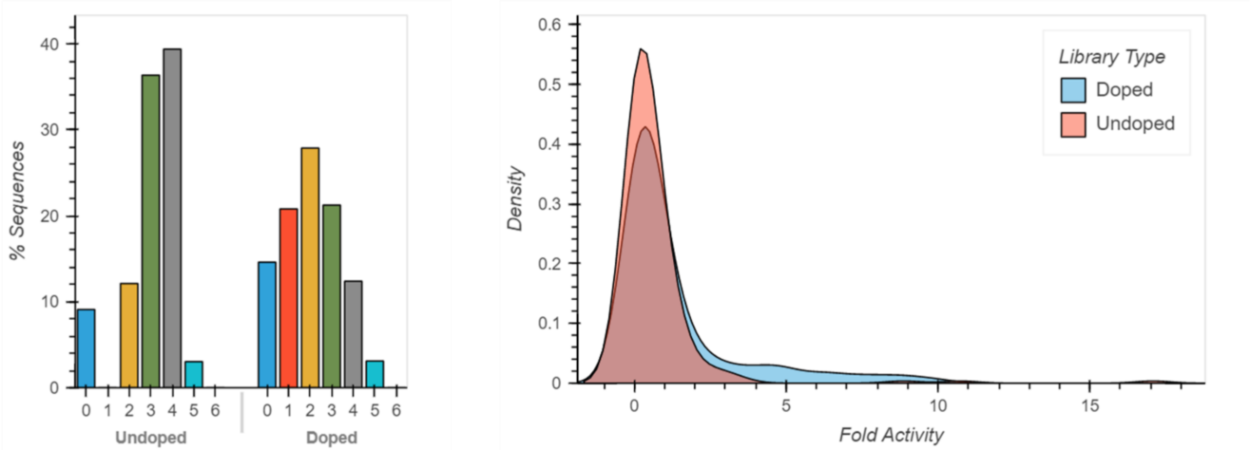

Supplementary Figure 4. Mutation load influence on cumulative activity changes. (left) Observed fraction of sequenced wells with a given number of mutations in undoped (n=33 wells) and doped (n=226 wells) plates, including parent control wells. **(right)** Kernel density estimate of the probability of a variant having a given fold change in total activity. These results show that primer doping reduces mutational load and samples a more active sequence space.

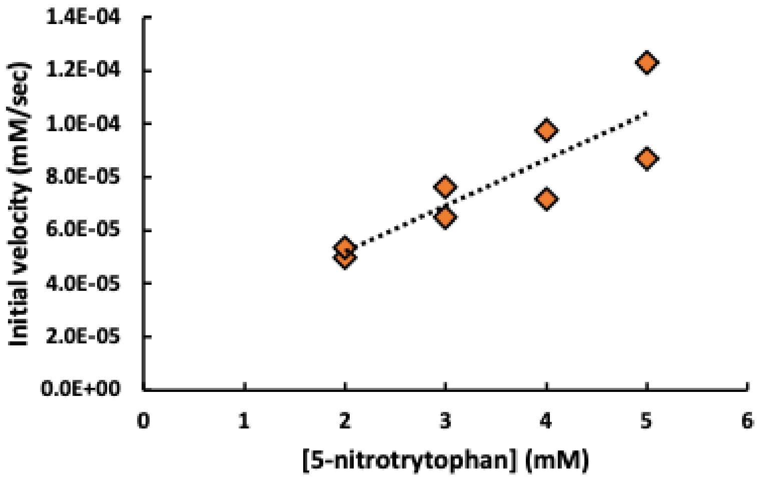

Supplementary Figure 5. Initial velocity measurements of wild-type *Rgn*TDC with 5-NO_2-Trp. The linear relationship indicates the $K_M > 5$ mM and the slope therefore corresponds to k_{cat}/K_M.

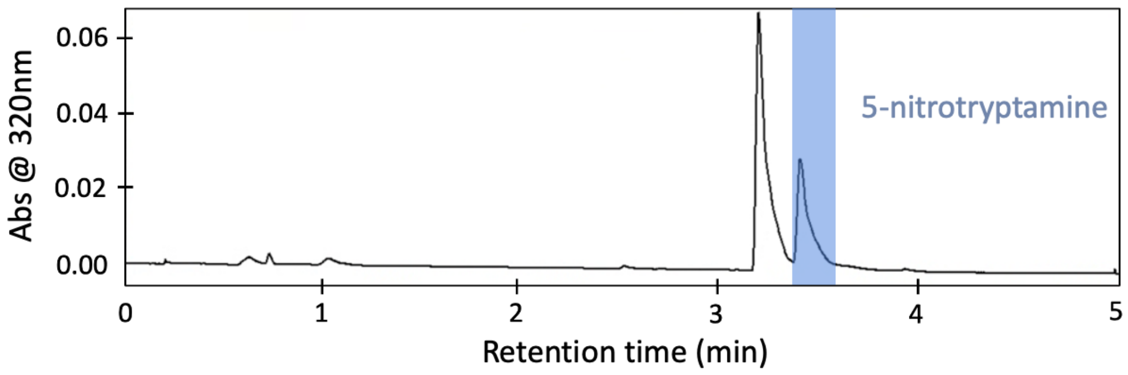

Supplementary Figure 6. Example of a UPLC-MS trace for the conversion of 5-nitrotryptophan to 5-nitrotryptamine.

Supplementary Tables

Supplementary Table 1. A) Confusion matrix and accuracy of trained logistic regression model. **B)** Confusion matrices and accuracies of trained logistic regression model on various active site mutational load data subsets.

A)

Global		Predicted		
		Inactive	Active	
Truth	Inactive	90	16	Accuracy
	Active	15	56	0.82

B)

0 Active Site Mutations		Predicted		
		Inactive	Active	
Truth	Inactive	0	0	Accuracy
	Active	0	2	1.00

1 Active Site Mutation		Predicted		
		Inactive	Active	
Truth	Inactive	0	2	Accuracy
	Active	0	14	0.88

2 Active Site Mutations		Predicted		
		Inactive	Active	
Truth	Inactive	8	12	Accuracy
	Active	0	36	0.79

3 Active Site Mutations		Predicted		
		Inactive	Active	
Truth	Inactive	40	1	Accuracy
	Active	10	3	0.80

4 Active Site Mutations		Predicted		
		Inactive	Active	
Truth	Inactive	34	1	Accuracy
	Active	5	1	0.85

5 Active Site Mutations		Predicted		
		Inactive	Active	
Truth	Inactive	8	0	Accuracy
	Active	0	0	1.00

Supplementary Table 2. Curated library used for variant validation. Within each plate section, 2 sterile control wells (D03, F03) and 3 parent control wells (C03, E03, G03) were included. For rescreening, this layout (A01-H04) was copied two additional times (A05-H08, A09-H12) to fill a 96-well plate.

			Mutation Profile					
Well	Name	Library Style	F98	V99	L339	W349	L355	I343
F01	V01	PostLogRM	L		M	S		N
G01	V02	Undoped			V	F	A	
H01	V03	PostLogRM	M		V	Y		N
A02	V04	Doped			V		A	
B02	V05	Doped			M	K	M	
F02	V06	PostLogRM				S	A	N
G02	V07	Undoped		F		R		
B04	V08	Undoped		F		A		
C04	V09	PostLogRM			V	T	M	N
D04	V10	Doped				V	M	
H04	V11	PostLogRM			M	C		N
A01	V12	Doped	R		V	Q		
B01	V13	PostLogRM	M		M			N
C01	V14	Doped	L	C		M		
D01	V15	PostLogRM	G		M			N
E01	V16	Doped				T	T	
C02	V17	Doped				K	A	
D02	V18	Undoped	T	G			S	
E02	V19	PostLogRM	T			C		N
H02	V20	Undoped	V	G	V		S	
A03	V21	PostLogRM	A		M			N
B03	V22	PostLogRM	M		M	P		N
H03	V23	Doped	I	S			A	
A04	V24	Doped	C	F		C	A	
E04	V25	Doped	C	F	M	A		
F04	V26	Doped	L	C		R	S	N
G04	V27	PostLogRM	T		M	F	A	N

Supplementary Table 3. Fold activities of tryptamine formation for all curated library variants (V01-V27) compared to parent when assayed against the indicated amino acid.

Name	Fold Activity											
	4-CN	4-OMe	5-NO_2	5-OEt	β-Me	4-Br	5-$CONH_2$	6-COOMe	7-I	Trp	Phe	Tyr
Parent	1.0	1.0	1.0	1.0	1.0	1.0	1.0	1.0	1.0	1.0	1.0	1.0
V01	0.1	0.4	5.6	22.4	0.3	0.1	0.2	0.2	0.0	0.5	0.0	0.0
V02	8.9	1.0	0.5	1.0	0.4	1.1	0.2	0.4	0.0	0.4	0.0	0.0
V03	0.1	0.6	8.6	7.3	0.3	0.2	0.9	2.3	0.4	1.0	0.0	0.0
V04	40.6	3.5	0.5	0.6	1.9	5.7	0.1	0.1	0.2	0.8	0.1	0.0
V05	0.1	0.4	23.4	7.5	0.3	0.2	0.2	0.2	0.0	0.3	0.0	0.0
V06	0.6	0.5	5.9	2.6	0.3	0.2	5.1	1.9	0.0	0.1	0.0	0.0
V07	0.1	0.4	12.9	1.1	0.3	0.1	0.1	0.0	0.0	0.5	0.0	0.0
V08	0.1	0.5	0.9	19.4	0.4	0.1	0.0	0.0	0.0	0.3	0.0	0.0
V09	0.1	0.5	11.0	6.3	0.3	0.1	0.8	1.6	0.0	0.2	0.0	0.0
V10	0.1	0.5	13.2	3.6	0.3	0.2	0.4	0.9	0.0	0.2	0.0	0.0
V11	0.2	0.7	4.1	21.5	0.3	0.2	0.6	0.7	0.1	0.8	0.1	0.0
V12	0.1	0.4	0.1	0.2	0.3	0.1	0.0	0.0	0.0	0.0	0.0	0.0
V13	0.2	0.8	1.7	2.0	0.3	0.4	0.5	0.9	0.7	1.0	0.1	0.1
V14	0.1	0.5	0.4	5.3	0.3	0.1	0.0	0.0	0.0	0.6	0.0	0.0
V15	0.1	0.7	0.4	0.8	0.3	0.1	0.1	0.3	0.0	0.8	0.0	0.0
V16	0.1	0.4	1.1	2.9	0.1	0.1	0.2	0.4	0.0	0.1	0.0	0.0
V17	0.2	0.4	2.0	0.1	0.3	0.2	0.0	0.0	0.0	0.0	0.0	0.0
V18	0.1	0.4	0.0	0.1	0.3	0.1	0.0	0.0	0.0	0.0	0.0	0.0
V19	0.1	0.4	0.1	2.5	0.1	0.1	0.0	0.0	0.0	0.1	0.0	0.0
V20	0.1	0.4	0.0	0.2	0.1	0.1	0.0	0.0	0.0	0.0	0.0	0.0
V21	0.2	0.7	1.3	1.5	0.3	0.2	0.5	0.6	0.4	1.0	0.1	0.0
V22	0.1	0.5	0.3	1.1	0.3	0.1	0.2	0.0	0.0	0.0	0.0	0.0
V23	0.1	0.5	0.0	0.1	0.3	0.1	0.0	0.0	0.0	0.0	0.0	0.0
V24	0.1	0.4	0.4	0.1	0.2	0.1	0.0	0.0	0.1	0.0	0.0	0.0
V25	0.1	0.5	0.0	0.6	0.3	0.1	0.0	0.0	0.0	0.0	0.0	0.0
V26	0.1	0.5	0.0	0.1	0.3	0.1	0.0	0.0	0.0	0.0	0.0	0.0
V27	0.2	0.4	0.0	0.1	0.3	0.1	0.0	0.0	0.0	0.0	0.0	0.0

Supplementary Materials and Methods

Chemicals and reagents were purchased from commercial suppliers (Sigma-Aldrich, VWR, Chem-Impex International, Alfa Aesar, Combi-blocks, Oakwood Products) at the highest quality available and used without further purification unless stated otherwise. Genes were purchased as gBlocks from Integrated DNA Technologies (IDT). E. coli cells were electroporated with a Bio-Rad MicroPulser at 2500 V. New Brunswick I26R, 120 V/60 Hz shaker incubators (Eppendorf) were used for cell growth. Cell disruption via sonication was performed with a Sonic Dismembrator 550 (Fisher Scientific) sonicator. Optical density measurements were collected using an optical density reader (Amersham Biosciences). Ultra-high pressure liquid chromatography-mass spectrometry (UPLC-MS) data were collected on an Acquity UPLC (Waters) equipped with an Acquity PDA and QDA MS detector using either a BEH C18 column (Waters) or an Intrada Amino Acid column (Imtakt). Utilized modified tryptophans/noncanonical amino acids were generated from their corresponding indoles using *Pf*TrpB^{2B9} as previously described.[1] Preparative column separations were performed on an Isolera One Flash Purification system (Biotage) using Sfar C18 columns.

Protein and DNA sequences

Protein Sequence of C-His-RgnTDC (Uniprot accession code: A7B1V0), mutated sites highlighted in red, active site in bold, I343 underlined, His tag in blue:

MSQVIKKKRNTFMIGTEYILNSTQLEEAIKSFVHDFCAEKHEIHDQPVVVEAKEHQEDKIKQIKIP
EKGRPVNEVVSEMMNEVYRYRGDANHPRFFS**FV**PGPASSVSWLGDIMTSAYNIHAGGSKLAPM
VNCIEQEVLKWLAKQVGFTENPGGVFVSGGSMANITALTAARDNKLTDINLHLGTAYISDQTHSS
VAKGLRIIGITDSRIRRIPTNSHFQMDTTKLEEAIETDKKSGYIPFVVIGTAGTTNTGSIDPLTEISAL
CKKHDMWFHIDGAYGASVLLSPKYKSLLTGTGLADSISWDAHKWLFQTYGCAMVLVKDIRNLF
HSFHVNPEYLKD**L**ENDIDNVNT**W**DIGME**L**TRPARGLKLWLTLQVLGSDLIGSAIEHGFQLAVWA
EEALNPKKDWEIVSPAQMAMINFRYAPKDLTKEEQDILNEKISHRILESGYAAIFTTVLNGKTVLRI
CAIHPEATQEDMQHTIDLLDQYGREIYTEMKKALEHHHHHH

DNA Sequence of C-His-RgnTDC, mutated sites highlighted in red, active site in bold, I343 underlined:

ATGTCGCAGGTCATTAAGAAAAAACGCAATACGTTTATGATTGGAACGGAGTACATCCTTAATT
CGACACAGTTAGAGGAGGCAATTAAGTCTTTCGTGCACGATTTTTGTGCGGAAAAACATGAG
ATCCATGATCAGCCCGTCGTTGTTGAAGCCAAGGAGCACCAGGAAGATAAAATTAAGCAGAT
CAAGATCCCTGAAAAAGGACGCCCAGTAAATGAGGTCGTGAGTGAGATGATGAATGAAGTTT
ACCGCTATCGCGGAGATGCGAACCACCCCCGTTTCTTCTCC**TTCGTT**CCGGGTCCAGCTTCGA
GCGTCTCCTGGCTTGGAGACATCATGACGAGTGCATATAATATCCATGCCGGAGGCAGTAAATT
GGCTCCCATGGTAAACTGTATTGAGCAAGAAGTGCTGAAGTGGTTGGCAAAGCAAGTGGGAT
TTACTGAAAATCCCGGCGGGGTGTTCGTCTCAGGTGGCTCGATGGCGAACATCACGGCGTTAA
CAGCAGCCCGTGACAATAAACTTACTGACATTAATTTGCATTTAGGAACGGCGTATATCAGCGA
CCAAACACACTCCAGTGTAGCCAAGGGGTTACGTATTATTGGCATCACCGACAGCCGTATTCG
CCGTATTCCCACTAATTCGCACTTCCAAATGGATACGACCAAGTTGGAGGAGGCCATTGAAAC
CGATAAAAAGAGTGGCTATATCCCGTTTGTAGTGATCGGAACCGCTGGCACGACTAATACAGG
ATCCATTGACCCATTAACGGAAATTTCTGCATTATGTAAAAAGCACGATATGTGGTTCCACATC
GACGGTGCGTATGGTGCCTCCGTATTGCTTAGTCCAAAATATAAGTCCCTTTTGACAGGAACA
GGATTAGCAGATAGTATTTCTTGGGATGCTCACAAATGGTTATTCCAGACGTATGGGTGCGCCA
TGGTATTGGTGAAGGACATCCGCAACCTGTTCCATTCCTTTCACGTTAACCCCGAATATCTGAA
AGAC**CTT**GAGAATGACATTGATAATGTCAATAC**GTG**GGATATTGGGATGGAG**TTA**ACACGTCC
GGCACGTGGGCTTAAACTTTGGCTGACCTTGCAGGTTCTTGGGTCCGACCTTATTGGGTCTGC
AATTGAGCACGGTTTCCAATTAGCGGTATGGGCGGAAGAAGCGCTGAATCCCAAAAAAGATT
GGGAAATTGTTAGCCCTGCCCAGATGGCGATGATTAATTTTCGCTACGCGCCTAAGGATTTAAC
CAAAGAGGAGCAGGACATCCTTAATGAAAAGATTTCGCATCGCATCTTGGAATCAGGCTATGC
CGCTATTTTTACTACTGTGCTGAATGGTAAGACAGTGTTACGCATTTGCGCGATTCACCCTGAG

GCTACTCAAGAGGATATGCAGCACACCATTGATCTGTTGGACCAATACGGTCGCGAGATCTATA CTGAAATGAAAAAGGCTCTCGAGCACCATCACCATCACCATTGA

Cloning, Expression, Purification, and Storage of *Rgn*TDC

A codon-optimized copy of the *Ruminococcus gnavus* tryptophan decarboxylase (*Rgn*TDC) gene was purchased as a gBlock from Integrated DNA Technologies. This DNA fragment was inserted into a pET22b vector (Addgene) by the Gibson Assembly method.[2] BL21 (DE3) *E. coli* cells were subsequently transformed with the resulting cyclized DNA product via electroporation. After 30 min of recovery in LB media at 37 °C, cells were plated onto LB plates with 100 μg/mL ampicillin (Amp) and incubated overnight. Single colonies were used to inoculate 5 mL TB + 100 μg/mL Amp (TB_{Amp}), which were grown overnight at 37 °C, 200 rpm. Expression cultures, typically 1 L of TB_{Amp} were inoculated from these starter cultures and shaken (180 rpm) at 37 °C. After 3.5 to 4 hours (OD_{600} > 1.5), the expression cultures were chilled on ice for 45 min. Expression was then induced with 1 mM IPTG, and the cultures were supplemented with 0.5 mM indole. Cultures were expressed overnight at 23 °C with shaking at 180 rpm. Cells were then harvested by centrifugation at 4300x*g* at 4 °C for 15 min. Cell pellets were frozen and stored at -20 °C until purification.

To purify *Rgn*TDC, cell pellets were thawed on ice and then resuspended in lysis buffer (50 mM potassium phosphate buffer (pH = 8.0), 1 mg/ml Hen Egg White Lysozyme (GoldBio), 0.2 mg/ml DNaseI (GoldBio), 1 mM $MgCl_2$, and 200 μM pyridoxal 5′-phosphate (PLP)). A volume of 4 mL of lysis buffer per gram of wet cell pellet was used. After 45 min of shaking at 37 °C, cells were sonicated with a ½ in. horn tip for 10 min (1 s on; 1 s off). The resulting lysate was then spun down at 75,000 x*g* to pellet cell debris. 1 mL bed volume of Ni-NTA beads (GoldBio), previously equilibrated in binding buffer (20 mM potassium phosphate (pH = 8.0), 20 mM imdazole) were added to the lysis supernatant and allowed to incubate for 1 h on ice with rocking. These beads then collected in a gravity column. The bed was washed with 4 column volumes binding buffer. Washing with higher concentrations of imidazole resulted in slow protein elution. TDC was eluted with 250 mM imidazole, 50 mM potassium phosphate buffer (pH = 8.0). Elution liquid was collected until the eluent transitioned from yellow (resulting from the TDC bound PLP cofactor) to colorless. The protein product was dialyzed to < 50 μM imidazole in 50 mM Tris-HCl buffer (pH = 8.0) or 50 mM potassium phosphate buffer (pH = 8.0). Purified enzyme was flash frozen in droplet form by pipetting enzyme dropwise into a crystallization dish filled with liquid nitrogen. The enzyme was transferred to a plastic conical and stored at -80 °C until further use. Frozen droplets were thawed at room temperature and centrifuged before use. The concentration of protein was determined by Bradford assay after freeze-thawing using bovine serum albumin for a standard concentration curve. Generally, this procedure yielded >100 mg TDC per L culture. Protein purity was analyzed by sodium dodecyl sulfate-polyacrylamide (SDS-PAGE) gel electrophoresis using 12% polyacrylamide gels.

Test reactions for substrate mixture design

Activity of *Rgn*TDC was tested on the following substrates individually and as a substrate pool: β-methyl-L-tryptophan, 4-cyano-L-tryptophan, 4-methoxy-L-tryptophan, 5-nitro-L-tryptophan, and 5-ethoxy-L-tryptophan. For the individual reactions, 10 mM tryptophan analog was added to a solution containing 500 μM PLP and 11.25 μM *Rgn*TDC (900 max TTNs) in 50 mM potassium phosphate buffer pH = 8.0. For the pooled reaction, 2 mM of each tryptophan analog was added to a solution containing 500 μM PLP and 11.25 μM *Rgn*TDC (900 max TTNs) in 50 mM potassium phosphate buffer pH = 8.0. Reactions were incubated for 16 h at 37 ºC, quenched via addition of 4 volumes of methanol, and spin-filtered to pellet protein prior to analysis via UPLC-MS. Conversion was estimated via analysis of substrate and product UV-vis peak areas for individual reactions. For the pooled reaction, relative product abundances were inferred from MS peak areas.

Library Construction

Generation of Original Recombination Library

Primers were purchased from Integrated DNA Technologies. Five active-site residues were targeted for mutagenesis, based on site-saturation mutagenesis library results[1,3]. The following degenerate codons were used for library construction: F98: DBK; V99: DBC; L339: DTG; W349: NDT + VHG + TGG; L355: DCA + ATG + TTG. The library was amplified first as two separate fragments and then combined via polymerase chain assembly (PCA) to form full-length *Rgn*TDC gene mutagenized at the sites of interest.[4] The corresponding genes were then inserted into a pET22b vector as described above, transformed into BL21(DE3) *E. coli* cells, and plated on LB + 100 µg/mL Amp agar plates. Individual colonies from these plates were used to inoculate 88 wells containing 700 µL TB_{Amp} in a 96-deep well plate. Five wells were inoculated with wild-type *Rgn*TDC (transformed at the same time as the library), two wells were inoculated with a non-TDC protein ($TrpB^{2B9}$) as a negative control, and one well was left as a sterile control. These plates were covered and allowed to grow in a 37 °C, 220 rpm shaker incubator overnight. The next day, 20 uL from each well was transferred to a new expression culture plate containing 630 uL TB_{Amp} per well and allowed to incubate at 37 °C, 220 rpm for 3 h. After incubation, the plates were placed on ice and allowed to cool for 30 min. Protein expression was then induced by addition of 50 uL TB_{Amp} containing 14 mM IPTG (final concentration 1 mM), and allowed to express overnight at 20 °C, 220 rpm. After expression and confirmation that sterile wells remained clear, cells were harvested by centrifugation at 4000 x*g* and 4°C, and the supernatant was poured off. Residual media was removed by firmly striking overturned plates onto a lab bench lined with paper towel. Pellets were stored at -20 °C until lysis and reaction screening. Additionally, 100 µL of each well from the overnight culture plate was mixed with 100 µL of sterile 50% glycerol and stored at -80 °C, done in duplicate. For each screened library plate, one of these glycerol stock plates was submitted for whole plate sequencing.

Note: In prior engineering campaigns with *Rgn*TDC, it was observed that cultures expressing the wild-type enzyme would grow to abnormally low density and, consequently, would yield less protein per volume culture.[3] We had hypothesized that expression of *Rgn*TDC led to concomitant decarboxylation of endogenous tryptophan, hampering cellular growth. It was discovered that inclusion of indole in the growth media to rescue growth via increasing and shortcutting L-tryptophan biosynthesis during protein expression was able to mitigate this loss in robust growth, providing good evidence towards our hypothesis. However, in the rounds of evolution disclosed in this publication, exogenous indole was omitted during protein expression. We hypothesized that this omission would place *Rgn*TDC variants under an implicit selection bias for diminished activity against endogenous amino acid, as reduced activity would lead to greater cell viability and catalyst yield, resulting in higher activity during screening.

Generation of Wild-Type Primer Doped Recombination Library

Wild-type codon primers for the five active-site positions were purchased from Integrated DNA Technologies. These were mixed with their corresponding mutageneic primers in the above original recombination library in a 3:2 wild-type:mutageneic primer ratio, leading to a predicted mutational rate of 2.5 mutations per variant. The remaining library production process matches that of the original recombination library process.

Generation of Regression-Guided Wild-Type Primer Doped Recombination Library

Regression-guided recombination primers for the five active-site positions were purchased from Integrated DNA Technologies. The following degenerate codons were used for library construction: F98: BKC + AYS + TGG + GCG; L339: DTG; I343N**:** AAT**;** W349: NDT + VYS + AAA + TGG; L355: DCA + ATG + TTG. For the parent gene of this library, the mutation I343N was fixed as a constant addition. Mutagenic primers were combined with wild-type primers (as in the original wild-type primer doped library construction) in a 7:3 mutageneic:wild-type primer ratio, leading to a predicted mutational rate of 3.0 mutations per variant. The remaining library production process matches that of the above libraries.

Screening of *Rgn*TDC Libraries

Library plates were thawed at room temperature, then 600 µL of lysis buffer was added to each well: 50 mM potassium phosphate buffer (pH = 8.0), 1 mg/mL Hen Egg White Lysozyme (GoldBio), 0.2 mg/mL DNaseI (GoldBio), 1 mM $MgCl_2$, and 300 µM pyridoxal 5′-phosphate (PLP). Plates were sealed with a rubber mat and vortexed to resuspend pelleted cells. After 1 h of shaking at 37 °C, the resulting lysate was then spun down at 4000 x*g* to pellet cell debris. 180 µL of the resulting supernatants were then added to 20 µL of a substrate mixture in a separate reaction plate. The substrate master mix contained 20 mM each Trp substrate dissolved in 50% MeOH, 40% H_2O, and 10% 1 M HCl. Final substrate concentrations are as follows: 2 mM β-methyl-L-tryptophan, 2 mM 4-cyano-L-tryptophan, 2 mM 4-methoxy-L-tryptophan, 2 mM 5-ethoxy-L-tryptophan, and 2 mM 5-nitro-L-tryptophan. Reactions were incubated at 37 °C for 4 h, and then 100 µL reaction solution was quenched via addition of 200 µL acetonitrile with vortexing. Quenched reaction plates were then centrifuged at 4000 x*g* for 10 min to pellet aggregated protein. 200 µL of the quenched reaction mixture supernatant was filtered into a 96-well plate for UPLC-MS analysis. Data were collected on an Acquity UPLC equipped with an Acquity QDA MS detector (Waters) using an 50 mm x 2 mm Intrada Amino Acid column (Imtakt) for separation of analytes. Product m/z ion counts were used to assess product formation from the reaction mixture. After activity screening, plates were submitted for whole plate sequencing, and full-length sequences were reconstructed and scored for validity using in-house Python tools.

Logistic Regression Modelling

All logistic regression was done using Python[5] 3.11 and the SciPy package.[6] Raw data files were exported from the Waters Empower software and integrated using in-house Python tools. Individual product fold activity values for variants were calculated by dividing the apparent MS ion count peak integrations against the average parent well area. For variants with multiple observations, these fold-activity values were averaged before labeling. These individual fold-activity measurements were then averaged to give the final "average fold-activity change" metric used for labelling (where wild-type activity corresponds to 1). Initial labeling thresholds for retention of function and mutational load distribution analysis were chosen as >0.3 average fold activity for "low activity" and >1.5 average fold activity for "high activity." For sequence featurization, mutations were encoded using the following concatenated one-hot mutation encoding map, where sampling of wild-type at a given position is represented as all 0's. Additionally, a one-hot encoding of the number of active site mutations present in a variant was appended to model the influence of mutational load.

F98X											V99X					L399X		I343X	W349X																			L355X				Active site mutations				
A	C	G	I	L	M	R	S	T	V	W	A	C	F	G	S	M	V	N	A	C	D	E	F	G	H	I	K	L	M	N	P	Q	R	S	T	V	Y	A	M	S	T	1	2	3	4	5

Area under curve (AUC) analysis using leave-one-out cross-validation (LOOCV) was used to determine the influence of active/inactive labeling thresholds on model performance. A final labeling threshold of 0.45 was chosen (where variants with an average fold-activity change less than 0.45 were labeled as inactive) for final model production. Receiver operating characteristic (ROC) analysis using LOOCV of the final model indicated a probability threshold of 0.48 produced the most accurate model, with an accuracy of 0.82 and F1 score of 0.78. Confusion matrices for each level of active site mutational load were generated to assess model accuracy dependence on mutational load (Supplementary Table 1), showing a mostly agnostic model. The weight matrix of the model (Supplementary Table 2) was then assessed to determine strongly deleterious mutations for removal.

Validation of Curated Variants

27 total variants were selected from each stage of mutagenesis based on sequence diversity, activity diversity, and overall average fold-activity changes compared to parent (Supplementary Table 3) These variants were compiled into a 96-well plate in triplicate to enable screening of three individual substrates in parallel. Production of cell lysates for screening was done in the same fashion as the original library screening protocol. Reaction plates were made by adding 20 µL of 100 mM substrate in a 50% MeOH,

40% H_2O, 10% 1 M HCl mixture, followed by addition of either 180 µL of lysate for substrates with known low (< 1000 TTN) parent activity or 160 µL 50 mM KP_i pH 8.0 and 20 µL lysate for substrates with known high (> 10,000 TTN) parent activity. These reactions were conducted and processed as in the original library screening protocol.

Michaelis-Menten analysis of *Rgn*TDC variants

*Rgn*TDC variants were thawed on ice from storage at -20 °C and then centrifuged at 15,000 xg for 5 min to pellet aggregated protein. The supernatant was diluted in 50 mM potassium phosphate buffer (pH = 8.0) such that the enzyme concentration was 10x the final reaction concentration. 5-nitrotryptophan was solubilized in 50% MeOH, 40% H_2O, and 10% 1 M HCl. In this mixture, the solubility limit of 5-nitrotryptophan was 60 mM. Reaction conditions used 0.5 – 6 mM 5-nitrotryptophan and 100 equivalents of PLP cofactor relative to TDC in 50-100 mM potassium phosphate buffer (pH = 8.0). Final enzyme concentrations were 5 uM for variant V05 and 50 µM for wild-type. Reactions were conducted in 1.5 mL Eppendorf tubes with a total volume of 350 uL. At various time points after addition of RgnTDC, 100 uL aliquots of the reaction solution were quenched in 100 uL acetonitrile and diluted with 150 – 200 uL H_2O. Quenched reactions were filtered prior to UPLC-MS injection of the supernatant. Enzymatic activity was quantified by integrating the substrate and product UV absorbance peaks at 320 nm. Initial velocities for V05 were fit using the modified Michealis-Menten equation to allow for cooperative binding.

Synthesis of Amino Acid Substrates

The following amino acid substrates were obtained from previous work and as described by McDonald et al. 2019[3]: 4-bromo-L-tryptophan, 4-cyano-L-tryptophan, 4-methoxy-L-tryptophan, 5-nitro-L-tryptophan, 7-iodo-L-tryptophan, β-methyl-L-tryptophan.
5-ethoxy-L-tryptophan and 5-carboxamido-L-tryptophan were obtained as described by McDonald and Higgins et al. 2022.[1]

6-carboxymethyltryptophan

To a 100 mL pressure vial was added 183.1 mg (1.0 mmol) 6-methyl-carboxylate indole, 220 mg (2.1 mmol) L-serine, 1 mL MeOH (2% final v/v), 400 µL 20 mM PLP (final = 160 µM), and 48 mL 50 mM potassium phosphate buffer pH = 8.0. $TrpB^{2B9}$ was then added to a concentration of 14 µM (0.07% mol catalyst, 1500 TTNs). The flask was then heated to 75 °C for 24 h. Water was evaporated via rotary evaporation to a volume of ~10 mL. The resultant slurry was injected onto a Sfär 12 g C18 cartridge (Biotage), and product containing peaks were identified via UV-vis absorbance and/or LC-MS analysis. Product containing peaks were pooled and the solvent evaporated before transfer to pre-tared vials. An off-white powder corresponding to 6-carboxymethyl-L-tryptophan. was obtained via lyophilization of 34 mg in 12% yield. **^{1}H NMR** (400 MHz, MeOD) **δ** 8.10 (s, 1H), 7.80 – 7.69 (m, 2H), 7.40 (s, 1H), 3.91 (s, 3H), 3.79 (dd, *J* = 8.6, 4.3 Hz, 1H), 3.46 (dd, *J* = 15.0, 4.2 Hz, 1H), 3.16 (dd, *J* = 15.2, 8.7 Hz, 1H).

NMR

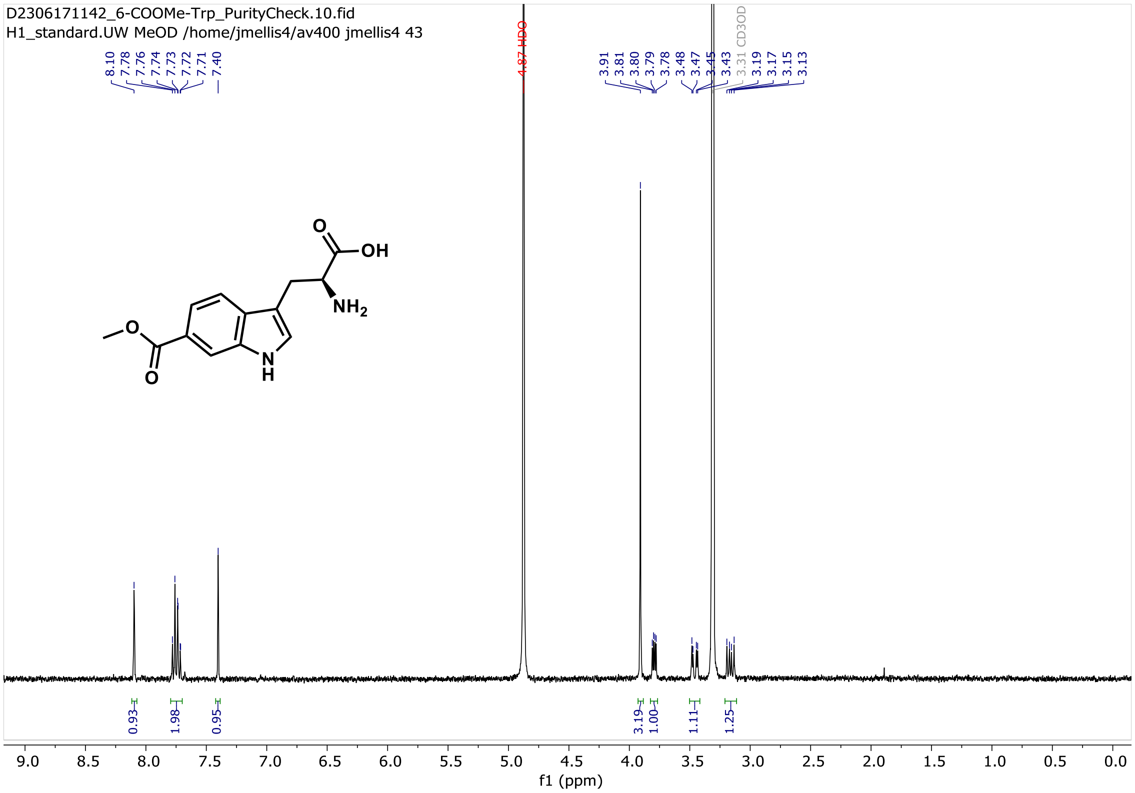

^{1}H NMR MeOD 400 MHz

Supplementary References

1. McDonald, A. D., Higgins, P. M. & Buller, A. R. Substrate multiplexed protein engineering facilitates promiscuous biocatalytic synthesis. *Nat Commun* **13**, 5242 (2022).

2. Gibson, D. G. Enzymatic Assembly of Overlapping DNA Fragments. in 349–361 (2011). doi:10.1016/B978-0-12-385120-8.00015-2.

3. McDonald, A. D., Perkins, L. J. & Buller, A. R. Facile in Vitro Biocatalytic Production of Diverse Tryptamines. *ChemBioChem* **20**, 1939–1944 (2019).

4. TerMaat, J. R., Pienaar, E., Whitney, S. E., Mamedov, T. G. & Subramanian, A. Gene synthesis by integrated polymerase chain assembly and PCR amplification using a high-speed thermocycler. *J Microbiol Methods* **79**, 295–300 (2009).

5. Van Rossum, G. & Drake, F. L. *Python 3 Reference Manual*. (CreateSpace, Scotts Valley, CA, 2009).

6. Virtanen, P. *et al.* SciPy 1.0: Fundamental Algorithms for Scientific Computing in Python. *Nat Methods* (2020) doi:https://doi.org/10.1038/s41592-019-0686-2.
